# Divergent Connectivity Changes in Gray Matter Structural Covariance Networks in Subjective Cognitive Decline, Amnestic Mild Cognitive Impairment, and Alzheimer’s Disease

**DOI:** 10.3389/fnagi.2021.686598

**Published:** 2021-08-16

**Authors:** Zhenrong Fu, Mingyan Zhao, Yirong He, Xuetong Wang, Jiadong Lu, Shaoxian Li, Xin Li, Guixia Kang, Ying Han, Shuyu Li

**Affiliations:** ^1^School of Biological Science and Medical Engineering, Beijing Advanced Innovation Center for Biomedical Engineering, Beihang University, Beijing, China; ^2^Department of Neurology, Tangshan Gongren Hospital, Tangshan, China; ^3^Department of Neurology, Xuanwu Hospital of Capital Medical University, Beijing, China; ^4^School of Electrical Engineering, Yanshan University, Qinhuangdao, China; ^5^Measurement Technology and Instrumentation Key Laboratory of Hebei Province, Qinhuangdao, China; ^6^School of Information and Communication Engineering, Beijing University of Posts and Telecommunications, Beijing, China; ^7^Biomedical Engineering Institute, Hainan University, Haikou, China; ^8^Center of Alzheimer’s Disease, Beijing Institute for Brain Disorders, Beijing, China; ^9^National Clinical Research Center for Geriatric Disorders, Beijing, China

**Keywords:** structural covariance network, subjective cognitive decline, structural MRI, default mode network, amnestic mild cognitive impairment, Alzheimer’s disease

## Abstract

Alzheimer’s disease (AD) has a long preclinical stage that can last for decades prior to progressing toward amnestic mild cognitive impairment (aMCI) and/or dementia. Subjective cognitive decline (SCD) is characterized by self-experienced memory decline without any evidence of objective cognitive decline and is regarded as the later stage of preclinical AD. It has been reported that the changes in structural covariance patterns are affected by AD pathology in the patients with AD and aMCI within the specific large-scale brain networks. However, the changes in structural covariance patterns including normal control (NC), SCD, aMCI, and AD are still poorly understood. In this study, we recruited 42 NCs, 35 individuals with SCD, 43 patients with aMCI, and 41 patients with AD. Gray matter (GM) volumes were extracted from 10 readily identifiable regions of interest involved in high-order cognitive function and AD-related dysfunctional structures. The volume values were used to predict the regional densities in the whole brain by using voxel-based statistical and multiple linear regression models. Decreased structural covariance and weakened connectivity strength were observed in individuals with SCD compared with NCs. Structural covariance networks (SCNs) seeding from the default mode network (DMN), salience network, subfields of the hippocampus, and cholinergic basal forebrain showed increased structural covariance at the early stage of AD (referring to aMCI) and decreased structural covariance at the dementia stage (referring to AD). Moreover, the SCN seeding from the executive control network (ECN) showed a linearly increased extent of the structural covariance during the early and dementia stages. The results suggest that changes in structural covariance patterns as the order of NC-SCD-aMCI-AD are divergent and dynamic, and support the structural disconnection hypothesis in individuals with SCD.

## Introduction

Alzheimer’s disease (AD), beginning with cognitive impairment, is the most common type of dementia, characterized by progressive and irreversible pathology with a long preclinical phase (Masters et al., 2015). Mild cognitive impairment (MCI) is the early symptomatic stage of AD, characterized by objective cognitive impairment, but largely preserves the daily functioning of individuals compared with dementia (Albert et al., 2011; Jack et al., 2018). Subjective cognitive decline (SCD) refers to the self-perceived worsening of cognitive ability, which is defined at the preclinical stage of AD and is independent of the neuropsychological tests (Jessen et al., 2014, 2020). The majority of individuals with SCD will not show sustained cognitive decline or progress to AD (Jessen et al., 2020) because the associations between self-perceived cognitive decline and objective cognitive impairment are complex. However, most of the studies have shown that symptoms of SCD may represent the earliest alert of AD, and individuals with SCD are at higher risk for developing AD or MCI (Rabin et al., 2017; Jessen et al., 2020; Wang et al., 2020b). Early diagnosis and intervention to preserve cognitive function is an important way to combat AD (Livingston et al., 2017); thus, it is critical to investigate the associations among biomarkers of SCD, MCI, and AD to provide a better opportunity for an early therapy (Jessen et al., 2020).

Reliable markers are crucial for diagnosis, intervention, and therapy in neurodegenerative diseases (Gao et al., 2020; Yang et al., 2020). For instance, the Aβ/Tau/neurodegeneration in AD-related disease (Jack et al., 2016), the “Hot cross bun” in multiple system atrophy with cerebellar ataxia (Zhu et al., 2021), and the motor abnormalities and α-synuclein in Parkinson’s disease (PD) (Xie et al., 2019) have been proven to be potential biomarkers for an early detection of these diseases. Focusing on AD pathology, both the accumulation of amyloid-β (Aβ) in plaques and aggregation of the protein tau in neurofibrillary tangles are biomarkers that can be used to monitor the progression of AD (Jack et al., 2016). Moreover, the initial locations of Aβ deposition are in the frontal lobes, temporal lobes, hippocampus, and limbic system; and pathologic tau originates in the medial temporal lobes and hippocampus (Braak et al., 2011; Braak and Del Tredici, 2015; Masters et al., 2015). Studies based on neuroimaging have described hippocampal atrophy as an effective biomarker in patients with AD (Zhao et al., 2019; Wang et al., 2020b) and patients with MCI (Jack et al., 2010), and individuals with SCD (Cantero et al., 2016; Zhao et al., 2019). In addition, studies based on the resting-state functional magnetic resonance imaging (rs-fMRI) techniques have revealed that a specific set of brain regions (including the posterior cingulate cortex, anterior medial prefrontal cortex, medial temporal lobe, lateral temporal cortex, and inferior parietal lobule) forms a functional network associated with the resting states (Buckner et al., 2008; Yeo et al., 2011; Montembeault et al., 2016), named as the default mode network (DMN). With regard to the neurodegeneration within the DMN, reduced gray matter (GM) volume in DMN regions in patients with AD (Liu et al., 2011) and patients with MCI (Liu et al., 2011; Tosun et al., 2011), and individuals with SCD (Hafkemeijer et al., 2013) has been found in multiple studies. It is worth noting that pathologic tau and Aβ accumulation in the cholinergic nucleus basalis emerged early in AD (Arendt et al., 1988; Mesulam et al., 2004), and volume reductions in the basal forebrain were observed in patients with AD (Kilimann et al., 2014) and individuals with SCD (Scheef et al., 2019). Collectively, the pathology of Aβ/tau/neurodegeneration in regions of the DMN, hippocampus, and basal forebrain has been investigated but much remains to be learned about the variations in coordination with other regions in normal controls (NCs) and those in SCD, amnestic mild cognitive impairment (aMCI), and AD.

Mapping whole-brain GM correlations with seed regions to construct a GM structural covariance network (SCN) based on structural magnetic resonance imaging (sMRI) has been proposed to investigate the covariance in GM density (Mechelli et al., 2005; Alexander-Bloch et al., 2013; Evans, 2013). Although the biological basis of the SCN is not very clear, there are many hypotheses, such as a result of mutually trophic influences (Mechelli et al., 2005), common experience-related plasticity (Mechelli et al., 2005), common neurodevelopmental blueprints for axonal guidance and neuronal migration (Zielinski et al., 2010), or a combination of these factors (Seeley et al., 2009). However, the SCN technique has been used in many studies, such as those examining development (Zielinski et al., 2010), sex differences in healthy adults (Mechelli et al., 2005), brain plasticity in adults (Guo et al., 2020), and connectivity alterations in patients with MCI (Wang et al., 2018) and patients with AD (Seeley et al., 2009; Montembeault et al., 2016; Li et al., 2019a). In studies of AD, decreased structural associations were observed within the DMN, and increased structural associations were shown within the salience network (SN) and executive control network (ECN) (Montembeault et al., 2016; Li et al., 2019a), which is partially in line with functional network studies (Seeley et al., 2009; Zhou et al., 2010). Moreover, SCNs seeded from subfields of the hippocampus in patients with MCI showed an increased extent of structural association compared with NCs (Wang et al., 2018). It is worth noting that increased functional connectivity in the DMN was observed in individuals with SCD (Hafkemeijer et al., 2013). However, the pattern changes in SCNs as the order of NC-SCD-aMCI-AD are still poorly known. This information may provide a better understanding of the underlying neuropathological mechanisms of SCD and the association between SCD- and AD-related diseases at the network level.

In the present study, we employed the SCN to explore changes in structural connectivity in specific large-scale networks as the order of NC-SCD-aMCI-AD. We defined 10 seed regions at three levels: (1) spheres anchored in the DMN, SN, and ECN; (2) anatomical regions of the bilateral anterior and posterior hippocampus; and (3) two anatomical subregions of the basal forebrain. Our results indicated that the trajectory of change patterns in SCNs along NC-SCD-aMCI-AD potentially provides structural covariance insight into better understanding of the progressive mechanism of cognitive decline due to AD-related pathology.

## Materials and Methods

### Participants

In the present study, 35 individuals with SCD, 43 patients with aMCI, and 41 patients with AD were recruited from the memory clinic of the Neurology Department of XuanWu Hospital, Capital Medical University, China. Then, 42 NC individuals were enrolled from local communities in Beijing, China. This study was performed in accordance with the rule of ethics of the Medical Research Ethics Committee in Xuanwu Hospital, and every subject gave their written informed consent to participate. The sample size was calculated by the analysis of covariance (ANCOVA) model in G^∗^Power 3.1.9.7 (Faul et al., 2007). The power (1-β) was 80%, α was 0.05, the effect size was 0.35, and the number of groups and covariates was 4. This calculation rendered a total sample size of 142, and 161 is larger than 142. The standard clinical assessments mainly included medical history investigations, neurological examinations, and neuropsychological tests. Cognitive tests included the Montreal Cognitive Assessment (MoCA, Beijing version) (Lu et al., 2011), auditory verbal learning test (AVLT) (namely, three memory tests: the AVLT immediate recall, the AVLT-delayed recall, and the AVLT recognition), the clinical dementia rating (CDR) (Morris, 1993), the Hamilton Depression Rating Scale (HAMD), the Activities of Daily Living (ADL) Scale, the Hachinski Ischemic Scale, and the Center for Epidemiologic Studies Depression Scale (Dozeman et al., 2011). In addition, the volunteers received a neuropsychological evaluation from two neurologists, each with more than 2 years of clinical experience in neurology.

The diagnostic criteria for individuals with SCD were based on the conceptual framework within the context of AD research proposed by the Subjective Cognitive Decline Initiative (Jessen et al., 2014), were described in our previous study (Fu et al., 2021), and included the following: (1) self-perceived memory decline without changes in other cognitive domains (last within 5 years); (2) feeling of worse performance than others of the same age group; (3) MoCA scores in the normal range (threshold was determined based on the different levels of education: primary school or below >19, secondary schooling >22, and university >24); (4) only one of the two memory tests (AVLT-delayed recall and AVLT recognition) was abnormal (one SD below NC performance); and (5) CDR score was 0. The patients with aMCI were identified with the Petersen criteria (Petersen, 2004), which included the following: (1) memory decline confirmed by an informant; (2) objective memory impairment measured by the MoCA and AVLT adjusted for education years (1.5 SD below NC performance); (3) CDR score of 0.5; (4) exclusion of subjects with other types of MCI, such as subcortical vascular MCI; and (5) exclusion of subjects with memory impairment of sufficient severity to affect the activities of daily living of the subject. The patients with AD were diagnosed based on the criteria of the National Institute of Aging-Alzheimer’s Association (NIA-AA) (Sperling et al., 2011) for clinically probable AD, which included the following: (1) symptoms consistent with the diagnostic criteria for dementia; (2) brain atrophy in the hippocampus based on sMRI; (3) gradual onset lasting more than 6 months rather than a sudden attack; and (4) CDR scores equal to 1 or higher.

The exclusion criteria for all subjects in the present study were as follows: (1) HAMD scores higher than 24 and a score on the Center for Epidemiologic Studies Depression Scale higher than 21; (2) the Hachinski Ischemic Scale in the abnormal range (higher than 4); (3) left-handedness; (4) impaired executive, visual, or auditory functions; (5) cognitive function decline due to non-AD neurological diseases (e.g., brain tumor, brain injury, PD, encephalitis, and normal pressure hydrocephalus); (6) history of stroke; (7) history of alcohol or drug abuse/addiction within 2 years; (8) large-vessel disease; (9) any other systemic diseases or uncertainty preventing the completion of the project; and (10) frequent head motion that may influence the quality of MRI data. The main demographic and clinical characteristics of the subjects are summarized in Table 1.

**TABLE 1:** Demographic characteristics and neuropsychological data.

	NC Group (42)	SCD Group (35)	aMCI Group (43)	AD Group (41)
Age (y)	64.24 ± 6.16	64.54 ± 7.29	67.47 ± 10.03	68.88 ± 7.86
Sex (men/women)	15/27	15/20	21/22	17/24
Education (y)	11.17 ± 5.61	11.83 ± 3.67	10.44 ± 4.96	9.68 ± 4.71
eTIV (* 10^6^ mm^3^)	1.40 ± 0.12	1.44 ± 0.14	1.47 ± 0.16	1.40 ± 0.13
MoCA	26.02 ± 2.95	25.26 ± 2.27	19.67 ± 4.28^aaabbb^	13.10 ± 5.46^aaabbbccc^
AVLT immediate recall	9.32 ± 1.94	8.54 ± 1.82	5.84 ± 1.34^aaabbb^	3.53 ± 1.58^aaabbbccc^
AVLT delayed recall	10.43 ± 2.31	8.86 ± 2.78^a^	3.19 ± 2.81^aaabbb^	0.98 ± 1.60^aaabbbccc^
AVLT recognition	12.07 ± 2.13	11.37 ± 2.20	6.58 ± 4.28^aaabbb^	3.51 ± 3.08^aaabbbccc^

*NC, normal control; aMCI, amnestic mild cognitive impairment; SCD, subjective cognitive decline; AD, Alzheimer’s disease; MoCA, Montreal Cognitive Assessment (Beijing version); AVLT, auditory verbal learning test; eTIV, estimated total intracranial volume.*

*The values represent the mean ± standard deviation.*

*^*a*^Denotes a significant difference between the NC group and other groups (^*a*^*p* < 0.05, ^*aa*^*p* < 0.01, and ^*aaa*^*p* < 0.001).*

*^*b*^Indicates a significant difference between the SCD group and the other groups (^*b*^*p* < 0.05, ^*bb*^*p* < 0.01, and ^*bbb*^*p* < 0.001).*

*^*c*^Represents a significant difference between the aMCI and other groups (^*c*^*p* < 0.05, ^*cc*^*p* < 0.01, and ^*ccc*^*p* < 0.001).*

### Image Acquisition

All T1-weighted images were acquired with a 3.0 T Siemens system (Magnetom Trio Tim; Erlangen, Germany) by a 3D sagittal magnetization-prepared rapid gradient echo (MPRAGE) sequence at the Department of Radiology, Xuanwu Hospital, Capital Medical University, Beijing, China. The parameters were as follows: TR = 1,900 ms; TE = 2.2 ms; TI = 900 ms; flip angle = 9°; FOV = 22.4 cm × 25.6 cm; matrix size = 448 × 512; number of slices = 176; and slice thickness = 1 mm (Zhao et al., 2019; Fu et al., 2021).

### Image Preprocessing

The non-uniformity intensity (N3) correction was first performed on anatomical T1 images by using FreeSurfer (version 6.0) (Fischl, 2012). After N3 correction, the images were analyzed with the CAT12 toolbox^1^. The pipeline in CAT12 includes removing noise with a spatial-adaptive non-local means denoizing filter (Manjón et al., 2010), segmenting the brain tissues into GM, white matter, and cerebrospinal fluid by local adaptive segmentation, partial volume estimation, and adaptive maximum *a posteriori* techniques, normalizing the images to a standard Montreal Neurological Institute (MNI) space by diffeomorphic anatomic registration through exponentiated Lie algorithm (Ashburner, 2007). Moreover, the GM images were modulated by Jacobian determinants to preserve the regional volume information. Finally, the GM images were smoothed in SPM12^2^ with a 6-mm (subregions of hippocampus and basal forebrain) and 12-mm (DMN, SN, and ECN) full-width at half-maximum Gaussian kernel.

### Definition of Seed Regions

Anatomical differentiation and functional segregation have been shown along the long axis of the hippocampus, and specializations arise out of differences between the anterior hippocampus and posterior hippocampus in large-scale network connectivity (Poppenk et al., 2013). In this study, the anterior hippocampus and posterior hippocampus were defined based on the previous studies (Poppenk et al., 2013; Li et al., 2018; Nordin et al., 2018), and we adopted the automated anatomical labeling (AAL) (Tzourio-Mazoyer et al., 2002) atlas for the segmentation of the hippocampus. In MNI coordinates, the anterior hippocampus masks from Y: −2 to −18 mm and the posterior hippocampus masks from Y: −24 to −42 mm. The seed regions representing functional large-scale networks were selected within the right entorhinal cortex (R EC) (MNI coordinates: 25, −9, and −28), left posterior cingulate cortex (L PCC) (MNI coordinates: −2, −36, and 35), right frontoinsular cortex (R FIC) (MNI coordinates: 38, 26, and −10), and right dorsolateral prefrontal cortex (R DLPFC) (MNI coordinates: 44, 36, and 20). These regions anchor the DMN (medial temporal lobe subsystem and midline core subsystem), SN, and ECN (Montembeault et al., 2016; Li et al., 2019a). Then, analyses of the contralateral regions of the R EC, L PCC, R FIC, and R DLPFC were performed. Finally, the subregions of the basal forebrain were defined by a basal forebrain atlas in MNI space that has been widely used in the previous studies (Kilimann et al., 2014; Scheef et al., 2019; Chen et al., 2021). The Ch4p (cholinergic system of the posterior nucleus basalis Meynert) and Ch1/2 (cholinergic system of combined clusters of the medial septum and the vertical limb of the diagonal band) with observed volume reductions in SCD (Scheef et al., 2019; Chen et al., 2021) and AD (Kilimann et al., 2014) were selected as seed regions. The volume of the hippocampus and basal forebrain subregions were represented by mean values of the modulated GM voxels within the masks in MNI space. For the functional large-scale network seed regions, 4-mm radius sphere masks were constructed by using the MarsBaR toolbox^3^, and the mean GM intensity was calculated.

### Structural Covariance Analysis

Multiple regression models combined with voxel-based statistical analysis were performed on the modulated GM images to investigate the structural covariance between seed regions and whole brain voxels in each group. The mean values extracted from the seed regions, age, sex, education years, and estimated total intracranial volume (eTIV) were used as covariates. We performed specific T contrasts to identify voxels expressing a positive correlation within each group (NC, SCD, aMCI, and AD). The resulting maps for each group were thresholded at *p* < 0.05, and the false discovery rate (FDR) was employed for multiple comparison correction. Cluster sizes larger than 100 voxels (337.5 mm^3^) were reported.

Furthermore, the between-group differences in structural covariance compared with the NC group were assessed by differences in slopes. We used a linear interaction model combined with dummy coding, and the mean values extracted from the seed regions, group, interaction term (group × mean values of seed regions), age, sex, education years, and eTIV were used as covariates. Specific T contrasts were established to map the significantly different structural covariance voxels in slopes between two groups, including positive and negative correlations. We set the threshold at *p* < 0.01 at the voxel level and *p* < 0.05 at the cluster level with two-tailed Gaussian random field (GRF) correction. Cluster sizes larger than 100 voxels (337.5 mm^3^) were reported. The coordinates of the peak intensity of the cluster within the scope of the AAL template were reported, except when there was only one cluster.

To investigate the correlation between the volume of structural covariance peak regions and clinical tests, we performed two-tailed partial correlation analysis within each group, which showed a significant difference in the structural covariance (Li et al., 2019a), and the effects of age, sex, and education years were ruled out (*p* < 0.05). The GM volume of the peak regions was extracted by spheres with a radius of 4 mm around the peak intensity coordinates.

Group differences in age, years of education, eTIV, and neuropsychological test scores (MoCA and AVLT) were evaluated by ANOVA (*p* < 0.05), and Bonferroni *post hoc* analysis was performed. The chi-square test was used to investigate the sex distribution.

## Results

### Demographics

There were no significant differences in age, sex, education years, or eTIV for each pair of groups. The AVLT-delayed recall scores were significantly lower in the SCD group compared with the NC group (*p* < 0.05). Moreover, all the neuropsychological test scores (MoCA and AVLT) in the aMCI group were significantly lower than those in the NC and SCD groups (*p* < 0.001). In addition, all the neuropsychological test scores (MoCA and AVLT) in the AD group were lower than those in the other groups (*p* < 0.001). The results are shown in Table 1 and Supplementary Figure 1.

### Patterns of Structural Covariance Within Groups

**FIGURE 1:**
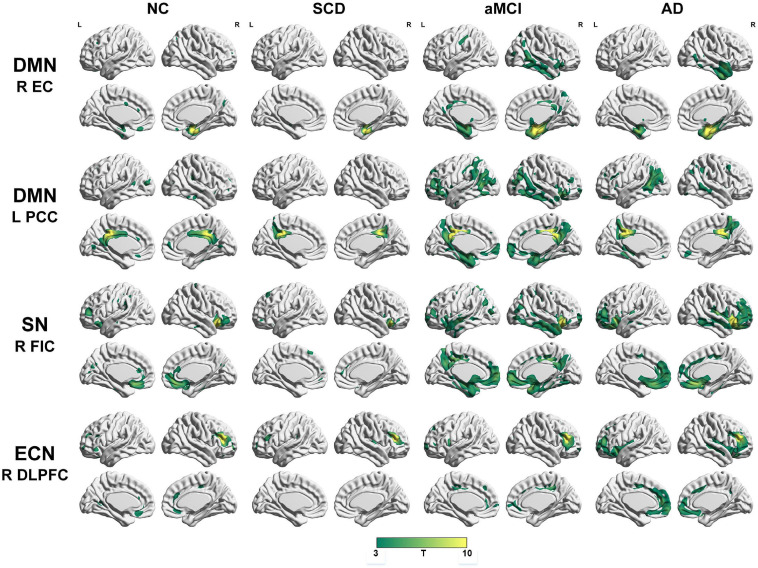
Structural covariance networks seeding from the default mode network, salience network, and executive control network within groups. T-statistic maps, *p* < 0.05, corrected by false discovery rate (FDR) with cluster size larger than 100 voxels. L, left; R, right; EC, entorhinal cortex; PCC, posterior cingulate cortex; DLPFC, dorsolateral prefrontal cortex; FIC, frontoinsular cortex; AD, Alzheimer’s disease; NC, normal control; aMCI, amnestic mild cognitive impairment; SCD, subjective cognitive decline.

**FIGURE 2:**
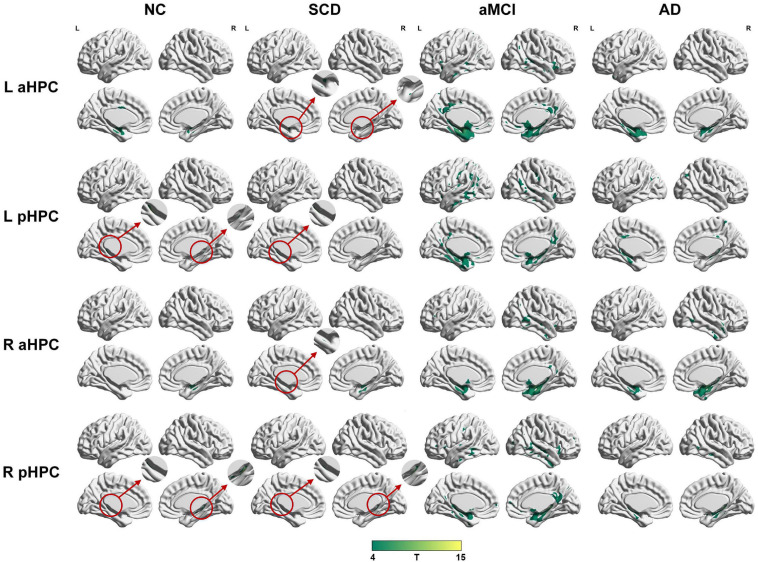
Structural covariance networks seeding from the anterior and posterior hippocampus within groups. T-statistic maps, *p* < 0.05, corrected by false discovery rate (FDR) with cluster size larger than 100 voxels. The small clusters are circled by the red circles, and they are enlarged. L, left; R, right; aHPC, anterior hippocampus; pHPC, posterior hippocampus; AD, Alzheimer’s disease; NC, normal control; aMCI, amnestic mild cognitive impairment; SCD, subjective cognitive decline.

**FIGURE 3:**
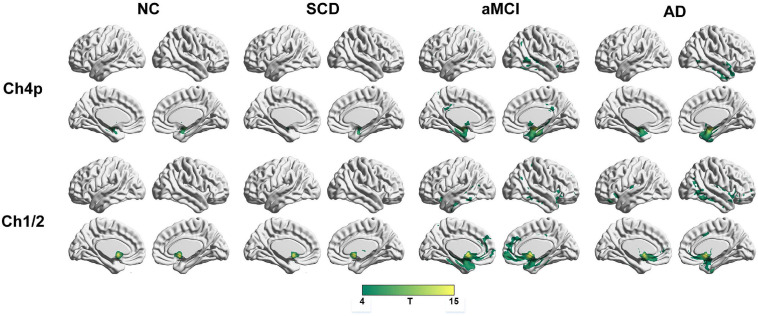
Structural covariance networks seeding from the Ch4p and Ch1/2 within groups. T-statistic maps, *p* < 0.05, corrected by false discovery rate (FDR) with cluster size larger than voxels. L, left; R, right; AD, Alzheimer’s disease; NC, normal control; aMCI, amnestic mild cognitive impairment; SCD, subjective cognitive decline.

To qualitatively compare the patterns of positive correlations across subjects within all groups, statistical maps are displayed in Figures 1–3, and the details are shown in Supplementary Tables 1–12. Regarding the DMN medial temporal subsystem, DMN midline core subsystem and SN, the aMCI group showed a greater extent of structural association than the other groups. In the ECN, the AD group exhibited an increased extent of structural association compared with the SCD, aMCI, and NC groups. The SCD group showed a decreased extent of structural covariance in both DMN subsystems, SN and ECN. In the DMN medial temporal subsystem, DMN midline core subsystem and SN, the number of clusters in the SCD group, aMCI and AD groups were decreased compared with the NC group. Regarding the bilateral anterior hippocampus and posterior hippocampus, the aMCI group showed a greater extent of structural covariance than the other groups. In all the subfields of the hippocampus, the subjects in the SCD group presented a decreased extent of structural covariance compared with the NC group. In the SCNs, seeded from subregions of the basal forebrain, both Ch4p and Ch1/2 showed a greater extent of structural covariance in the aMCI group than in the other groups. The results of contralateral seeds for the DMN subsystems, SN, and ECN, obtained by changing the sign on the x coordinate of each seed, are listed in Supplementary Tables 1–4. In addition, the results of other subregions of the basal forebrain are listed in Supplementary Tables 9–12.

### Significant Between-Group Differences in the Structural Covariance GM Network

**FIGURE 4:**
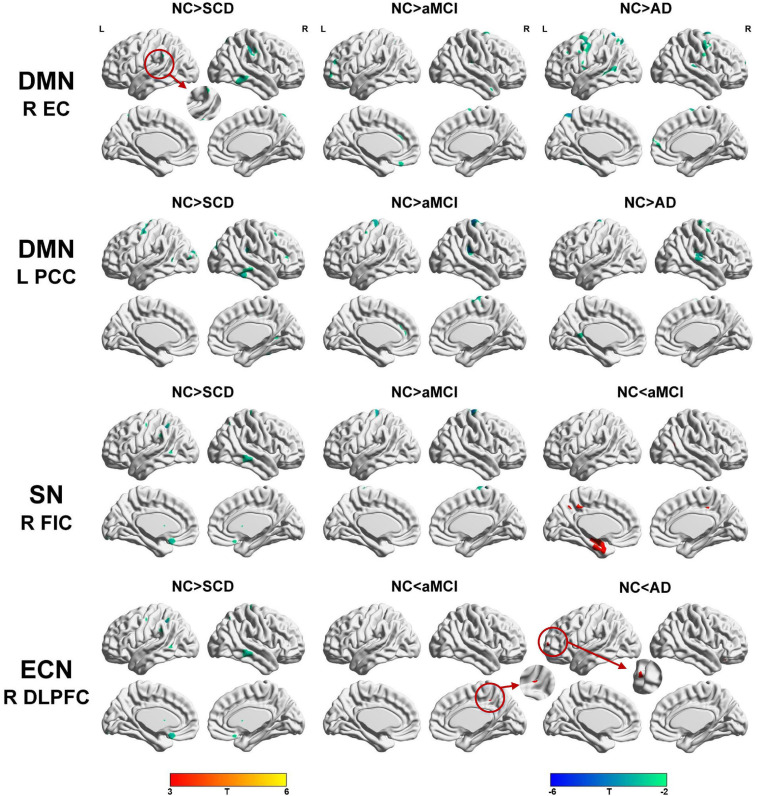
Between-group (NC-SCD; NC-aMCI; and NC-AD) differences in structural covariance networks seeding from the default mode network, salience network, and executive control network. T-statistic maps, *p* < 0.01 at the voxel level and *p* < 0.05 at the cluster level, two-tailed Gaussian random field (GRF) correction with cluster size larger than 100 voxels. The small clusters are circled by the red circles, and they are enlarged. L, left; R, right; EC, entorhinal cortex; PCC, posterior cingulate cortex; DLPFC, dorsolateral prefrontal cortex; FIC, frontoinsular cortex; AD, Alzheimer’s disease; NC, normal control; aMCI, amnestic mild cognitive impairment; SCD, subjective cognitive decline.

Regarding the DMN medial temporal subsystem, the subjects in the NC group showed increased strength in structural covariance compared with those in the SCD group in the right supramarginal gyrus (2,280 voxels) and in the left precentral gyrus compared with the aMCI (3,034 voxels) and AD (10,492 voxels) groups. Regarding the DMN midline core subsystem, the subjects in the NC group showed significantly increased strength in the structural covariance compared with those in the SCD group in the right inferior temporal gyrus (5,277 voxels), those in the aMCI group in the left middle frontal gyrus (7,638 voxels), and those in the AD group in the left precentral gyrus (1,743 voxels). Regarding the SN, the subjects in the NC group showed significantly increased strength in structural covariance compared with those in the SCD group in the left inferior parietal gyrus (2,289 voxels) and those in the aMCI group in the right precentral gyrus (1,541 voxels); the subjects in the NC group showed decreased strength in structural covariance in the right middle temporal gyrus compared with subjects in the aMCI group (3,646 voxels). Regarding the ECN, the subjects in the NC group showed significantly increased strength in structural covariance compared with those in the SCD group in the right inferior temporal gyrus (865 voxels); the subjects in the NC group showed decreased strength in structural covariance compared with those in the aMCI group in the right median cingulate (269 voxels) and those in the AD group in the right precuneus (934 voxels). The results are shown in Table 2 and Figure 4.

**TABLE 2:** Significant between-group (NC-SCD, NC-aMCI, and NC-AD) differences in structural covariance seeding from the DMN, SN, and ECN.

Seed	Contrast	Peak region	MNI coordinates			Extent	Peak intensity
					
			X	Y	Z		
R entorhinal cortex	NC > SCD	SupraMarginal_R	63	−24	43.5	2280	–3.9511
	NC > aMCI	Precentral_L	–7.5	−7.5	39	3034	–3.9178
	NC > AD	Precentral_L	–3.5	−6	43.5	10492	–4.5574
	NC < AD	ParaHippocampal_R	18	−1.5	–2.5	273	3.267
L posterior cingulate cortex	NC > SCD	Temporal_Inf_R	49.5	−36	–6.5	5277	–4.2714
	NC > aMCI	Frontal_Mid_L	–5.5	24	34.5	7638	–5.4367
	NC > AD	Precentral_L	–6.5	–6.5	72	1743	–3.7043
	NC < SCD	Precuneus_R	3	−51	45	357	3.2657
	NC < aMCI	Temporal_Mid_R	51	–58.5	16.5	431	3.3112
	NC < AD	Temporal_Mid_R	42	–52.5	19.5	1034	4.0244
R frontoinsular cortex	NC > SCD	Parietal_Inf_L	–34.5	−45	48	2289	–3.8531
	NC > aMCI	Precentral_R	15	−27	79.5	1541	–4.7806
	NC > AD	Precentral_R	15	−27	75	751	–3.8561
	NC < aMCI	Temporal_Mid_R	49.5	–46.5	6	3646	3.2717
	NC < AD	Supp_Motor_Area_R	12	−18	49.5	489	3.1579
R dorsolateral prefrontal cortex	NC > SCD	Temporal_Inf_R	54	−48	−27	865	–3.3482
	NC > AD	Calcarine_L	−15	–49.5	10.5	106	–3.0684
	NC < SCD	Occipital_Mid_L	−39	–67.5	1.5	313	3.4323
	NC < aMCI	Cingulum_Mid_R	7.5	−39	43.5	269	3.1672
	NC < AD	Precuneus_R	12	−57	40.5	934	3.602

*L, left; R, right; AD, Alzheimer’s disease; NC, normal control; aMCI, amnestic mild cognitive impairment; SCD, subjective cognitive decline; MNI, Montreal Neurological Institute.*

**FIGURE 5:**
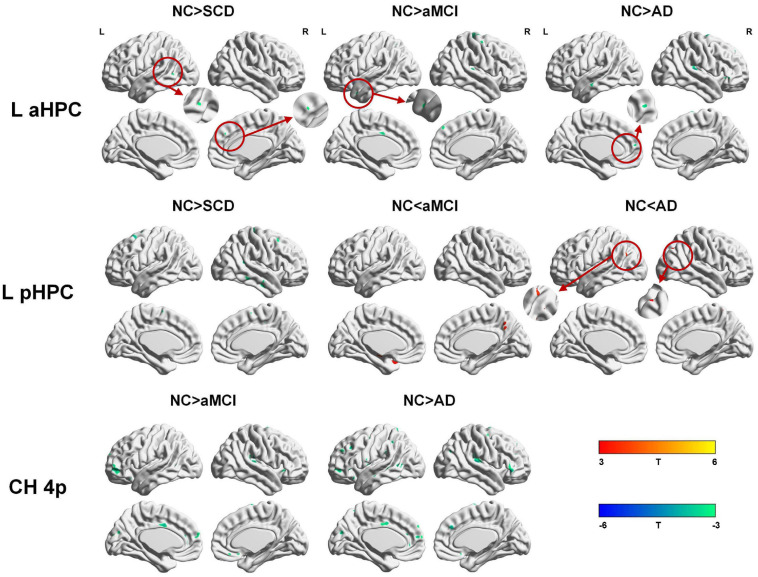
Between-group (NC-SCD; NC-aMCI; and NC-AD) differences in structural covariance networks seeding from the left anterior and posterior hippocampus and Ch4p. T-statistic maps, *p* < 0.01 at the voxel level and *p* < 0.05 at the cluster level, two-tailed GRF correction with cluster size larger than 100 voxels. The small clusters are circled by the red circles, and they are enlarged. L, left; R, right; aHPC, anterior hippocampus; pHPC, posterior hippocampus; AD, Alzheimer’s disease; NC, normal control; aMCI, amnestic mild cognitive impairment; SCD, subjective cognitive decline.

Within the SCN seeding from the left anterior hippocampus, decreased strength in structural covariance were observed in the SCD (left middle temporal gyrus; 313 voxels), aMCI (right precentral gyrus; 2,119 voxels), and AD (right superior temporal gyrus; 2,665 voxels) groups compared with the NC group. Moreover, within the SCN seeding from the left posterior hippocampus, decreased strength in structural covariance was shown in the SCD group (right middle temporal gyrus; 4,204 voxels) compared with the NC group; subjects in the NC group showed decreased structural covariance compared with those in the aMCI (right middle temporal gyrus; 1,656 voxels) and AD (left angular gyrus; 603 voxels) groups. Then, within the SCN seeding from the right anterior hippocampus, the subjects in the NC group showed increased strength in structural covariance compared with those in the SCD (right supramarginal gyrus; 1,307 voxels) and AD (left middle frontal gyrus; 1,900 voxels) groups. Within the SCN seeding from the right posterior hippocampus, the subjects in the NC group showed increased strength in structural covariance compared with those in the SCD (left superior frontal gyrus; 2,815 voxels) and AD (right parahippocampal gyrus; 3,511 voxels) groups. In addition, within the SCN seeding from the Ch4p, the subjects in the NC group showed increased strength in structural covariance compared with those in the aMCI (right superior temporal gyrus; 5,533 voxels) and AD (right median cingulate gyrus; 10,790 voxels) groups. In addition, within the SCN seeding from the Ch1/2, the subjects in the NC group showed increased strength in structural covariance compared with those in the SCD (left parahippocampal gyrus; 1,044 voxels) and AD (left superior frontal gyrus; 1,161 voxels) groups. The results are summarized in Tables 3, 4 and Figure 5. The results with the contralateral seeds for the DMN subsystems, SN and ECN, obtained by changing the sign on the x coordinate of each seed, are listed in Supplementary Tables 13–15, and the results of other subregions of the basal forebrain are listed in Supplementary Tables 19–21.

**TABLE 3:** Significant between-group (NC-SCD, NC-aMCI, and NC-AD) differences in structural covariance networks seeding from anterior and posterior hippocampi.

Seed	Contrast	Peak region	MNI coordinates			Extent	Peak intensity
					
			X	Y	Z		
L anterior hippocampus	NC > SCD	Temporal_Mid_L	–52.5	–49.5	0	313	–3.7864
	NC > aMCI	Precentral_R	43.5	−12	61.5	2119	–4.0192
	NC > AD	Temporal_Sup_R	40.5	−27	10.5	2665	–4.0872
	NC < aMCI	Hippocampus_L	–25.5	–13.5	–13.5	239	5.0774
	NC < AD	Precuneus_R	3	−48	60	411	3.6251
L posterior hippocampus	NC > SCD	Temporal_Mid_R	49.5	−48	15	4204	–5.1987
	NC > aMCI	Precentral_R	16.5	−27	73.5	878	–4.3176
	NC > AD	Frontal_Sup_L	−18	16.5	49.5	1193	–3.9886
	NC < aMCI	Temporal_Mid_R	48	–58.5	13.5	1656	4.5496
	NC < AD	Angular_L	−52.5	−69	30	603	4.1876
R anterior hippocampus	NC > SCD	SupraMarginal_R	58.5	–28.5	42	1307	–4.3683
	NC > aMCI	Temporal_Sup_R	57	–31.5	15	206	–4.1619
	NC > AD	Frontal_Mid_L	−28.5	28.5	36	1900	–4.4319
	NC < SCD	Occipital_Mid_L	−42	–73.5	1.5	123	3.788
	NC < AD	Insula_R	43.5	13.5	−7.5	382	4.2362
R posterior hippocampus	NC > SCD	Frontal_Sup_L	−16.5	22.5	63	2815	–4.5136
	NC > aMCI	Precentral_R	15	−27	75	571	–4.0688
	NC > AD	ParaHippocampal_R	33	−36	−4.5	3511	–4.4366
	NC < aMCI	Temporal_Mid_R	49.5	−60	13.5	570	3.9456

*L, left; R, right; AD, Alzheimer’s disease; NC, normal control; aMCI, amnestic mild cognitive impairment; SCD, subjective cognitive decline; MNI, Montreal Neurological Institute.*

**TABLE 4:** Significant between-group (NC-SCD, NC-aMCI, and NC-AD) differences in structural covariance networks seeding from the Ch4p and Ch1/2.

Seed	Contrast	Peak region	MNI coordinates			Extent	Peak intensity
					
			X	Y	Z		
Ch4p	NC > SCD	Temporal_Mid_L	–52.5	−51	1.5	133	–3.5685
	NC > aMCI	Temporal_Sup_R	55.5	−30	16.5	5533	–5.6571
	NC > AD	Cingulum_Mid_R	1.5	36	31.5	10790	–5.0984
	NC < SCD	Occipital_Mid_L	–40.5	−72	3	126	3.5079
Ch1/2	NC > SCD	ParaHippocampal_L	−30	−18	–22.5	1044	–4.1796
	NC > aMCI	Temporal_Sup_R	57	−30	15	279	–4.4193
	NC > AD	Frontal_Sup_L	−24	6	64.5	1161	–4.2794
	NC < AD	Temporal_Pole_Sup_R	55.5	16.5	–13.5	287	3.8021

*Ch4p, cholinergic system of posterior nucleus basalis Meynert; Ch1/2, cholinergic system of combined clusters of the medial septum and the vertical limb of the diagonal band; AD, Alzheimer’s disease; NC, normal control; aMCI, amnestic mild cognitive impairment; SCD, subjective cognitive decline; MNI, Montreal Neurological Institute.*

### Correlation Analysis Between Peak Cluster Volume and Cognitive Test Scores

We then performed partial correlation analysis between the peak cluster volumes with significant interaction effects and cognitive test scores within each group. The correlations were mainly located in the SCN seeding from DMN regions such as the hippocampus and posterior cingulate cortex. In the SCD group, the left anterior hippocampus-anchored (*r* = −0.351, *p* = 0.049) and posterior hippocampus-anchored (*r* = 0.505, *p* = 0.003) peak volumes (middle temporal gyrus) showed significant correlations with AVLT recognition scores. In the aMCI group, the peak volume in the hippocampus (left anterior hippocampus-anchored) significantly correlated with AVLT-delayed recall (*r* = 0.351, *p* = 0.027) and AVLT recognition (*r* = 0.456, *p* = 0.003) scores; the peak volume in the middle temporal gyrus (left posterior hippocampus-anchored) significantly correlated with AVLT recognition scores (*r* = 0.420; *p* = 0.007); the peak volume in the middle temporal gyrus (right posterior hippocampus-anchored) significantly correlated with AVLT recognition scores (*r* = 0.463; *p* = 0.003); the peak volume in the middle temporal gyrus (DMN midline core subsystem-anchored) significantly correlated with AVLT recognition scores (*r* = 0.401; *p* = 0.010); and the peak volume in the middle temporal gyrus (SN-anchored) significantly correlated with AVLT recognition scores (*r* = 0.376; *p* = 0.017). In the AD group, the peak volume in the precuneus showed a significant correlation with AVLT-delayed recall scores (*r* = 0.450, *p* = 0.005). The results of the correlation analyses are summarized in Supplementary Tables 22–24.

## Discussion

The present study aimed to investigate the AD-related changes in the GM in SCNs in individuals with SCD and the patients with aMCI and AD. Regarding the DMN and SN, the subjects in the aMCI and AD groups showed increased and decreased structural associations, respectively. Regarding the ECN, the subjects in the aMCI and AD groups exhibited linearly increased structural associations. Specifically, the SCNs anchored to the DMN, SN, and ECN decreased in the SCD group compared with the NC group. The pattern changes of SCNs seeding from the anterior hippocampus, posterior hippocampus, Ch4p, and Ch1/2 as the order of NC-SCD-aMCI-AD showed similar trends to the SCNs anchored to the DMN. However, the connectivity strength of the SCNs seeding from the DMN, SN, anterior hippocampus, posterior hippocampus, Ch4p, and Ch1/2 decreased in the individuals with SCD, aMCI, and AD compared with the NCs. In addition, the connectivity strength of the SCNs seeded from the ECN was increased in the patients with aMCI and AD. Our results suggest that the pattern changes in the SCNs as the order of NC-SCD-aMCI-AD are dynamic and divergent, which may provide evidence for disconnection in SCNs in individuals with SCD.

The results are partially consistent with previous studies showing changes in the DMN (Seeley et al., 2009; Zhou et al., 2010; Li et al., 2015; Chang et al., 2018; Xue et al., 2019), SN (Zhou et al., 2010; Li et al., 2015), and ECN (Weiler et al., 2014; Li et al., 2015) in patients with MCI and AD based on fMRI analysis. Moreover, the results in this study were generally in line with the previous studies based on SCNs, which observed changes in the DMN, SN, and ECN in patients with AD (Montembeault et al., 2016; Chang et al., 2018; Li et al., 2019a) and MCI (Shu et al., 2018). The possible underlying mechanism is that Aβ/tau/neurodegeneration pathological processes originate and concentrate in the DMN regions (Masters et al., 2015), the connectivity strength within the DMN is weakened, and more tissue is needed for the functional compensation. However, functional compensation by a large-scale network was shown in the SN and ECN due to AD pathology in our results. In our opinion, performing functional compensation is a more complex process, indicating that there may be multiple ways to participate in this process, not just compensation by large-scale networks. Although the results from the SCN analysis do not perfectly overlap the results with the functional network, many researchers agree that SCN analysis is an effective tool to investigate the topological organization of the brain and serves as a measure of network integrity in the cross-sectional group studies (Zielinski et al., 2010; Alexander-Bloch et al., 2013; Montembeault et al., 2016; Li et al., 2019a).

SCNs seeded from the anterior hippocampus and posterior hippocampus have been used to investigate the effects of aging (Li et al., 2018; Nordin et al., 2018), memory (Nordin et al., 2018), plasticity (Guo et al., 2020), and sex (Persson et al., 2014). However, the connectivity changes of SCNs induced by AD-related pathology seeding in the anterior hippocampus and posterior hippocampus remain poorly understood. Although the hippocampus belongs to the DMN medial temporal lobe subsystem, the function of the hippocampus is more focused on memory. With specialization along the long axis, the hippocampus was divided into two anatomical structures, the anterior hippocampus and the posterior hippocampus. Moreover, long-range connections between the anterior hippocampus and the perirhinal cortex, amygdala, hypothalamus, temporal lobe, insula, and prefrontal cortex; and long-range connections between the posterior hippocampus and the parahippocampal cortex, cingulate cortex, cuneus, precuneus, prefrontal cortex, and parietal lobe have been confirmed in humans (Poppenk et al., 2013). Our results suggested that the pattern changes in SCNs seeded from the anterior hippocampus and posterior hippocampus were similar to those of SCNs seeded from the DMN. However, a greater extent of structural covariance was shown in the anterior hippocampus than in the posterior hippocampus in all groups, which was consistent with a previous study (Li et al., 2018). The possible mechanism is that the neurodegenerative diseases were similar to the accelerated aging, and the age-related functional connectivity strength in healthy adults between the posterior hippocampus and DMN was stronger than the connectivity between the anterior hippocampus and DMN (Damoiseaux et al., 2016), while the connectivity changes in the DMN induced by AD-related pathology may have a greater impact on the connections between the posterior hippocampus and DMN compared with the connections between the anterior hippocampus and DMN. In addition, a previous study based on SCNs reported that structural connectivity between the hippocampus and DMN regions was limited to the anterior hippocampus, although these discrepancies may have been due to methodological differences (Li et al., 2018).

Atrophy in the cholinergic basal forebrain has been observed in advanced age (Grothe et al., 2012), individuals with SCD (Scheef et al., 2019; Chen et al., 2021), and patients with AD (Grothe et al., 2012; Kilimann et al., 2014). Specifically, a functional network analysis seeding from the anterior basal forebrain observed positive functional connectivity of the anterior basal forebrain mainly located in the DMN; and connectivity of the posterior basal forebrain mainly located in the SN in individuals with SCD (Chiesa et al., 2019). In the present study, the structural connectivity of Ch4p and Ch1/2 was mainly located in the DMN medial temporal subsystem in the individuals with SCD. Thus, the discrepancies may be due to methodological differences and different delineation protocols. However, structural connectivity of the Ch4p and Ch1/2 in the patients with aMCI located in both the medial temporal subsystem and midline core subsystem of the DMN and SN was observed. In addition, the pattern changes of SCN seeding from the Ch4p were similar to the pattern changes of SCN seeding from the R EC, and the pattern changes of SCN seeding from the Ch1/2 were similar to the pattern changes of SCN seeding from the L PCC as the order of NC-SCD-aMCI-AD. In addition, the pattern changes of SCN seeding from the Ch4p were similar to the pattern changes of SCN seeding from the hippocampus as the order of NC-SCD-aMCI-AD. As described in a previous study, a significant association between the volume in the Ch4p and right precuneus hypometabolism was shown in SCD (Scheef et al., 2019). In summary, the atrophy of Ch4p has the potential to be a neurodegeneration biomarker in the early stages of AD.

Regarding the DMN, studies based on the functional network showed that connectivity within the DMN was dysfunctional due to the pathology of AD (Seeley et al., 2009; Zhou et al., 2010), and studies based on the SCN showed that the structural connectivity within the DMN medial temporal subsystem was disrupted due to the pathology of AD (Montembeault et al., 2016; Li et al., 2019a). In the present results, the structural covariance within the DMN medial temporal subsystem in patients with AD was increased compared with that in NCs. Genetic effects may be a reason (Bi et al., 2019; Chang et al., 2019; Huang et al., 2019; Li et al., 2019b; Tao et al., 2019), and studies focusing on the effect of Bcl-2 rs956572 (Chang et al., 2018) and MTHFR C677T (Chang et al., 2017) based on SCN showed that homozygotes and heterozygotes exhibited different SCN patterns, although the subjects were all diagnosed with AD. Moreover, different diagnostic criteria and acquisition parameters may be a reason, and the patients with AD in the Alzheimer’s disease Neuroimaging Initiative database^4^ are at early stages of AD (CDR > 0.5), but the patients with AD in the present study included those in the early, mid-term, and late stages of AD (CDR ≥ 1). However, our results are partially in line with those of an SCN-based study, in which the structural covariance in patients with AD increased compared with NCs within the DMN midline core subsystem (Li et al., 2019a). Specifically, although the scope of structural covariance in the AD-related patients increased compared with the NCs, the connectivity strength weakened in the AD-related patients compared with the NCs. The weakened connections in our results in individuals with SCD, aMCI, and patients with AD were mainly located in the precentral gyrus, temporal lobe, prefrontal cortex, and parietal lobe. Conclusively, we speculate that the structural covariance of the DMN showed structural hyperconnectivity at the aMCI stage, and then hypoconnectivity was observed in the dementia stage.

The large-scale network referred to as the SN due to its consistent activation in response to emotionally significant internal and external stimuli showed altered function in AD-related patients (Zhou et al., 2010; Li et al., 2019a). Our results showed that the structural covariance of the SN increased in the AD-related patients compared with the NCs, which was consistent with a previous study (Montembeault et al., 2016). This result suggested that enhancement of connectivity in the SN may compensate for dysfunction in the DMN due to AD-related pathology (Zhou et al., 2010; Montembeault et al., 2016). Then, a linear increase in structural covariance of the ECN was observed with the progression through the NC-aMCI-AD continuum, which may support the hypothesis that AD is associated with opposing connectivity in the DMN and ECN (Zhou et al., 2010; Montembeault et al., 2016; Li et al., 2019a). In addition, we speculate that the ECN acts as a compensatory large-scale network for disconnections in the DMN due to AD pathology.

The SCNs seeded from the DMN, SN, ECN, anterior hippocampus, posterior hippocampus, Ch4p, and Ch1/2 in the SCD group showed a decreased extent of structural covariance compared with the NC group. However, the interaction model revealed that loss of connectivity strength of SCNs was observed within the DMN, SN, ECN, anterior hippocampus, posterior hippocampus, Ch4p, and Ch1/2 in the individuals with SCD. Regarding the ECN, the subjects with SCD showed enhanced connectivity strength in the middle occipital gyrus with a small cluster (313 voxels). In addition, a previous study based on the functional network revealed that increased functional connectivity in DMN regions was observed in individuals with SCD compared with NCs (Hafkemeijer et al., 2013). However, there are no reported studies based on SCN to explore SCD. Combined with a previous study (Hafkemeijer et al., 2013), the results in the present study suggested that structural associations decreased in individuals with SCD, and functional compensation was observed, but structural compensation was not found. These results are potential to indicate that individuals with SCD are at high risk of cognitive decline further.

Not only AD but also the other neurodegenerative diseases and cerebral small vessel disease (Zhu et al., 2019) may show cognitive decline at the early stage, such as progressive supranuclear palsy (Yang et al., 2021), cerebral autosomal dominant arteriopathy with subcortical infarcts and leukoencephalopathy (CADASIL) (Guo et al., 2021), and subcortical vascular MCI (Wang et al., 2018). Therefore, a specific biomarker is very important for disease diagnosis, intervention, and therapy. This study aimed to explore the imaging markers of SCD, aMCI, and AD based on sMRI at the network level. The results showed that the pattern changes in the SCNs as the order of NC-SCD-aMCI-AD are dynamic and divergent. In addition, the decreased extent of SCNs and the weakened connectivity strength of SCNs compared with NC are potential to be the imaging biomarkers for SCD. It is worth noting that the atrophy of the entorhinal cortex was observed both in patients with AD and PD (Jia et al., 2019). In the future, it will be interesting to investigate whether the SCNs seeding from the entorhinal cortex present distinct patterns in patients with AD and PD for understanding the pathology of two neurodegenerative diseases.

There were some limitations in the present study. First, the study was based on cross-sectional data. Although we examined NCs and those with SCD, aMCI, and AD to investigate the pattern changes of SCN, a further longitudinal study should be conducted. Indeed, a longitudinal study is more appropriate to investigate the pattern changes across time. Second, this study used SCN analysis based only on sMRI to explore the connectivity changes of large-scale networks, and a future study combined with a functional network based on fMRI should be done. The combination of multiple modality images may provide a better understanding of the mechanism of neurodegenerative diseases from both structural and functional sight. Third, there is a very limited neuropsychological battery in this dataset, and more neuropsychological tests should be included in our next dataset. Fourth, previous studies demonstrated that diabetes would affect cognition in patients with PD (Wang et al., 2020a), whether the diabetes would affect cognition in SCD is still poorly known. Moreover, cognitive decline may be induced not only by neurodegenerative diseases but also by mental state or physical frailty (Ma and Chan, 2020), such as depression and anxiety, and more information will be collected in our next cohort study. Finally, the relationship between neurocognitive function and neuropathogenesis is complex, a future study combined with integrated results of neuroimaging and the AD biomarkers such as Aβ and tau should be more persuasive.

## Conclusion

In the present study, we investigated the connectivity changes of GM SCNs in individuals with SCD, aMCI, and AD. A decreased extent of structural covariance and weakened structural connectivity strength were observed in individuals with SCD compared with NCs. Moreover, the divergent and dynamic connectivity changes of SCNs seeding from the DMN, SN, and ECN as the order of NC-SCD-aMCI-AD were shown in this study. Then, the patterns of SCN seeding from subregions of the hippocampus and basal forebrain were similar to those of SCN seeding from the DMN. In summary, the divergent trajectory of change patterns in SCNs along NC-SCD-aMCI-AD potentially provides structural covariance insight into better understanding the progressive mechanism of cognitive decline due to AD-related pathology at preclinical and early stages.

## Data Availability Statement

The raw data supporting the conclusions of this article will be made available by the authors, without undue reservation.

## Ethics Statement

The studies involving human participants were reviewed and approved by Ethics of the Medical Research Ethics Committee in Xuanwu Hospital. The patients/participants provided their written informed consent to participate in this study.

## Author Contributions

ZF and SyL were responsible for the conception and design of this study and wrote the first manuscript. ZF and XW performed the image preprocessing and experiments. YH and MZ performed the data acquisition. SyL reviewed and critiqued the manuscript. YrH, JL, and SxL assisted in drafting the manuscript. XL and GK reviewed and critiqued the manuscript. All authors contributed to the article and approved the submitted version.

## Conflict of Interest

The authors declare that the research was conducted in the absence of any commercial or financial relationships that could be construed as a potential conflict of interest.

## Publisher’s Note

All claims expressed in this article are solely those of the authors and do not necessarily represent those of their affiliated organizations, or those of the publisher, the editors and the reviewers. Any product that may be evaluated in this article, or claim that may be made by its manufacturer, is not guaranteed or endorsed by the publisher.

## Footnotes

1
http://dbm.neuro.uni-jena.de/cat/

2
https://www.fil.ion.ucl.ac.uk/spm/

3
http://marsbar.sourceforge.net/

4
http://adni.loni.usc.edu
