# Supplementary material for: Divergent Connectivity Changes in Gray Matter Structural Covariance Networks in Subjective Cognitive Decline, Amnestic Mild Cognitive Impairment, and Alzheimer’s Disease
Source: Front Aging Neurosci. 2021 Aug 16;13:686598. doi: 10.3389/fnagi.2021.686598 (PMC8415752; doi:10.3389/fnagi.2021.686598)
Supplement: Supplementary file 1 [file Data_Sheet_1.docx]

Supplementary Material


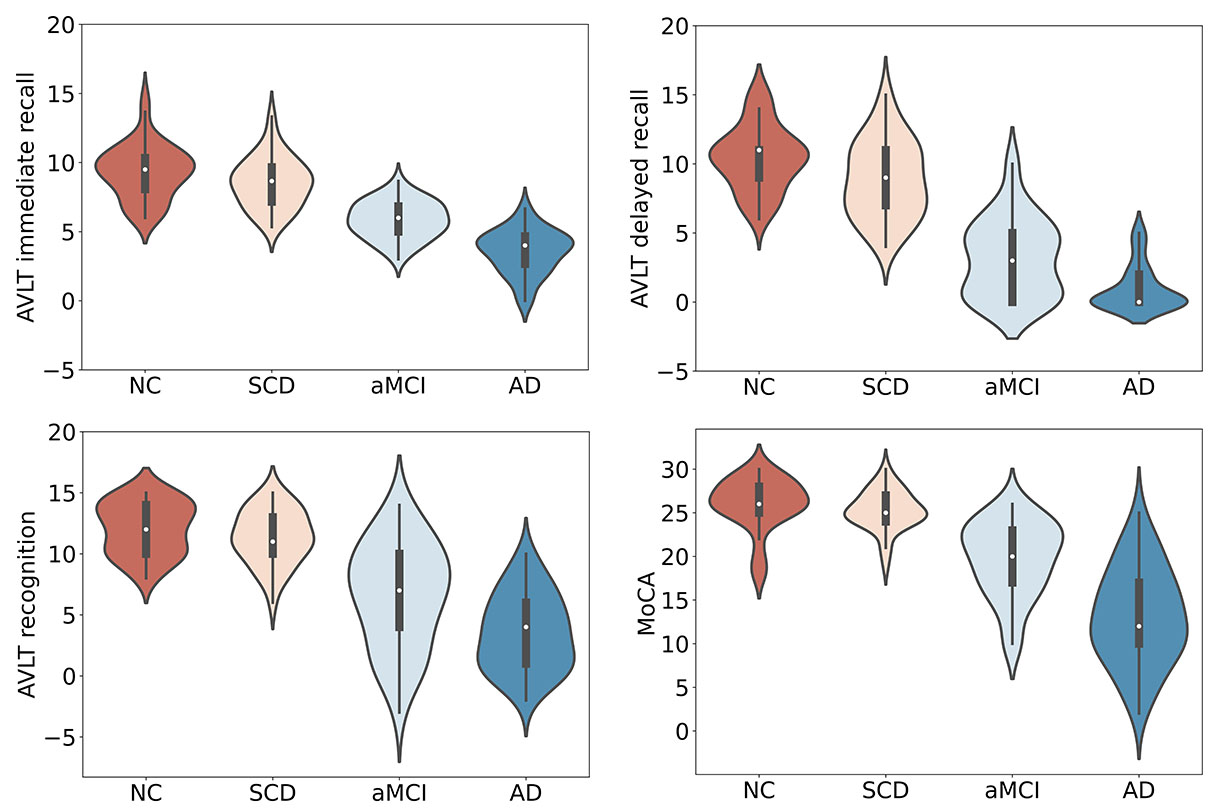


Supplementary Figure 1 The violin plots of the neuropsychological data of NC, SCD, aMCI and AD. NC, normal controls; aMCI, amnestic mild cognitive impairment; SCD, subjective cognitive decline; AD, Alzheimer’s disease; MoCA, Montreal Cognitive Assessment (Beijing version); AVLT, auditory verbal learning test.

**Supplementary Table 1.** Structural covariance networks seeding from DMN, SN, ECN and their contralateral regions in NC group.

| seed | AAL region | MNI coordinates | | | | Peak intensity | Extent |
| --- | --- | --- | --- | --- | --- | --- | --- |
|  |  | X | Y | | Z |  |  |
| L EC | ParaHippocampal_L | -24 | -9 | -28.5 | | 61.3455 | 5348 |
| L EC | ParaHippocampal_R | 31.5 | -12 | -27 | | 7.2483 | 1670 |
| R EC | Hippocampus_L | -25.5 | -13.5 | -21 | | 6.2836 | 3404 |
| R EC | ParaHippocampal_R | 25.5 | -9 | -28.5 | | 64.33 | 6549 |
| R EC | Rectus_L | -3 | 34.5 | -24 | | 5.1989 | 693 |
| R EC | Temporal_Pole_Sup_L | -43.5 | 27 | -24 | | 4.8806 | 503 |
| R EC | Occipital_Sup_R | 21 | -87 | 12 | | 5.7632 | 173 |
| R EC | Frontal_Sup_R | 28.5 | 55.5 | 18 | | 4.6125 | 586 |
| R EC | Cingulum_Ant_L | -6 | 30 | 19.5 | | 4.5963 | 230 |
| R EC | Frontal_Mid_L | -33 | 49.5 | 33 | | 4.5397 | 265 |
| R EC | Cuneus_R | 13.5 | -76.5 | 43.5 | | 5.0147 | 911 |
| R EC | Cingulum_Mid_L | -3 | 3 | 34.5 | | 4.6935 | 286 |
| R EC | Frontal_Mid_L | -31.5 | 28.5 | 34.5 | | 4.6438 | 206 |
| R EC | Precuneus_L | -13.5 | -57 | 61.5 | | 4.5495 | 110 |
| L PCC | Temporal_Inf_L | -49.5 | -25.5 | -18 | | 5.492 | 2750 |
| L PCC | Frontal_Mid_R | 34.5 | 55.5 | 25.5 | | 5.9475 | 5330 |
| L PCC | Insula_R | 39 | 21 | -7.5 | | 4.199 | 904 |
| L PCC | Cingulum_Mid_L | -1.5 | -36 | 34.5 | | 74.7911 | 26300 |
| L PCC | Insula_L | -36 | -13.5 | 3 | | 4.7798 | 849 |
| L PCC | Frontal_Med_Orb_L | -10.5 | 57 | -1.5 | | 3.8993 | 133 |
| L PCC | Occipital_Mid_L | -36 | -84 | 12 | | 5.9482 | 1499 |
| L PCC | Frontal_Mid_L | -27 | 27 | 34.5 | | 4.9527 | 578 |
| L PCC | Frontal_Mid_R | 28.5 | 30 | 42 | | 4.8192 | 583 |
| L PCC | Frontal_Sup_L | -19.5 | 15 | 49.5 | | 4.1255 | 137 |
| L PCC | Precentral_L | -21 | -16.5 | 63 | | 4.7851 | 516 |
| R PCC | Temporal_Inf_L | -49.5 | -25.5 | -18 | | 5.6507 | 2792 |
| R PCC | Temporal_Mid_R | 49.5 | -37.5 | -13.5 | | 6.3938 | 3279 |
| R PCC | Rectus_L | -7.5 | 31.5 | -18 | | 4.5674 | 1076 |
| R PCC | Insula_R | 36 | 16.5 | -15 | | 4.2202 | 711 |
| R PCC | Cingulum_Mid_R | 1.5 | -36 | 34.5 | | 71.5448 | 15563 |
| R PCC | Lingual_L | -21 | -45 | -4.5 | | 4.9224 | 352 |
| R PCC | Insula_L | -34.5 | -15 | 4.5 | | 5.0088 | 887 |
| R PCC | Frontal_Mid_R | 33 | 55.5 | 24 | | 5.9238 | 3803 |
| R PCC | Occipital_Mid_L | -34.5 | -84 | 12 | | 5.872 | 1264 |
| R PCC | Frontal_Mid_L | -27 | 28.5 | 33 | | 4.9948 | 506 |
| R PCC | Frontal_Mid_R | 28.5 | 30 | 42 | | 4.8292 | 476 |
| R PCC | Precentral_L | -18 | -16.5 | 63 | | 4.5627 | 334 |
| L FIC | Frontal_Inf_Orb_L | -39 | 25.5 | -10.5 | | 55.0191 | 138519 |
| L FIC | Lingual_R | 19.5 | -75 | -10.5 | | 4.0201 | 466 |
| L FIC | Lingual_L | -16.5 | -67.5 | -7.5 | | 3.7881 | 268 |
| L FIC | Cuneus_L | -4.5 | -85.5 | 16.5 | | 5.1478 | 2876 |
| L FIC | Occipital_Mid_R | 30 | -90 | 9 | | 3.8656 | 241 |
| L FIC | Parietal_Inf_L | -33 | -58.5 | 54 | | 4.8019 | 1670 |
| L FIC | Parietal_Sup_R | 24 | -49.5 | 73.5 | | 4.1704 | 1156 |
| R FIC | SupraMarginal_L | -49.5 | -39 | 33 | | 5.3598 | 7504 |
| R FIC | ParaHippocampal_R | 34.5 | -19.5 | -25.5 | | 6.0418 | 1813 |
| R FIC | Temporal_Inf_R | 63 | -19.5 | -19.5 | | 4.3457 | 1013 |
| R FIC | Cuneus_L | -1.5 | -70.5 | 28.5 | | 5.2679 | 2135 |
| R FIC | Temporal_Sup_R | 57 | -21 | 10.5 | | 5.6977 | 842 |
| R FIC | Frontal_Inf_Orb_R | 39 | 25.5 | -10.5 | | 57.7097 | 34056 |
| R FIC | Cingulum_Mid_R | 1.5 | 3 | 33 | | 3.8596 | 158 |
| R FIC | Frontal_Mid_L | -25.5 | 30 | 33 | | 3.9992 | 274 |
| R FIC | Cingulum_Mid_L | -7.5 | -30 | 34.5 | | 4.0057 | 258 |
| R FIC | Precentral_R | 12 | -27 | 73.5 | | 5.2454 | 1407 |
| R FIC | Supp_Motor_Area_R | 1.5 | 13.5 | 67.5 | | 4.0533 | 125 |
| L DLPFC | Frontal_Inf_Tri_L | -43.5 | 36 | 19.5 | | 60.9814 | 118951 |
| L DLPFC | Temporal_Inf_L | -46.5 | -51 | -24 | | 4.5676 | 2269 |
| L DLPFC | Occipital_Sup_R | 21 | -93 | 15 | | 3.9829 | 422 |
| L DLPFC | Precentral_L | -37.5 | -3 | 40.5 | | 3.8508 | 200 |
| L DLPFC | Precentral_L | -33 | -16.5 | 49.5 | | 4.7927 | 1579 |
| L DLPFC | Precuneus_R | 4.5 | -48 | 55.5 | | 3.7439 | 212 |
| L DLPFC | Postcentral_R | 25.5 | -45 | 63 | | 3.7737 | 191 |
| R DLPFC | Frontal_Mid_R | 43.5 | 36 | 19.5 | | 76.6169 | 14279 |
| R DLPFC | Frontal_Sup_Orb_L | -21 | 52.5 | -4.5 | | 6.3456 | 3362 |
| R DLPFC | Frontal_Inf_Tri_L | -40.5 | 33 | 1.5 | | 6.0187 | 2246 |
| R DLPFC | Temporal_Sup_L | -58.5 | -40.5 | 16.5 | | 4.138 | 156 |
| R DLPFC | Precentral_L | -31.5 | -18 | 51 | | 4.968 | 459 |
| R DLPFC | Cingulum_Mid_R | 12 | -24 | 40.5 | | 4.932 | 836 |

Abbreviations: L, left; R, right; EC, entorhinal cortex; PCC, posterior cingulate cortex; DLPFC, dorsolateral prefrontal cortex; FIC, frontoinsular cortex; DMN, default mode network; SN, salience network; ECN, executive control network; NC, normal control.

**Supplementary Table 2.** Structural covariance networks seeding from DMN, SN, ECN and their contralateral regions in SCD group.

| seed | AAL region | MNI coordinates | | | | | Peak intensity | Extent |
| --- | --- | --- | --- | --- | --- | --- | --- | --- |
|  |  | X | Y | | | Z |  |  |
| L EC | ParaHippocampal_L | -25.5 | | -9 | -28.5 | | 49.4701 | 1837 |
| R EC | ParaHippocampal_R | 24 | | -9 | -28.5 | | 63.2289 | 5071 |
| R EC | Hippocampus_L | -25.5 | | -16.5 | -16.5 | | 5.7479 | 281 |
| L PCC | Cingulum_Mid_L | -1.5 | | -36 | 34.5 | | 72.087 | 5987 |
| R PCC | Cingulum_Mid_R | 1.5 | | -36 | 34.5 | | 78.2457 | 5738 |
| L FIC | Frontal_Inf_Orb_L | -39 | | 25.5 | -10.5 | | 47.1672 | 2935 |
| L FIC | Frontal_Inf_Orb_R | 42 | | 43.5 | -3 | | 5.6652 | 611 |
| R FIC | Frontal_Inf_Orb_R | 37.5 | | 25.5 | -10.5 | | 46.1441 | 5859 |
| R FIC | Insula_L | -33 | | 19.5 | -16.5 | | 4.4798 | 105 |
| R FIC | Frontal_Mid_Orb_L | -39 | | 45 | -7.5 | | 4.9212 | 348 |
| R FIC | Frontal_Med_Orb_L | -10.5 | | 49.5 | -10.5 | | 4.9201 | 336 |
| R FIC | Insula_L | -46.5 | | 10.5 | -4.5 | | 5.8733 | 757 |
| R FIC | Frontal_Sup_Medial_R | 13.5 | | 51 | 6 | | 5.5477 | 1114 |
| R FIC | Frontal_Sup_L | -13.5 | | 25.5 | 49.5 | | 6.4702 | 1802 |
| L DLPFC | Frontal_Inf_Tri_L | -43.5 | | 36 | 19.5 | | 70.7066 | 8995 |
| L DLPFC | Temporal_Sup_R | 46.5 | | -21 | 1.5 | | 5.4324 | 1200 |
| L DLPFC | Frontal_Mid_R | 40.5 | | 36 | 22.5 | | 6.4188 | 5644 |
| L DLPFC | Thalamus_L | -4.5 | | -10.5 | 12 | | 4.5151 | 148 |
| L DLPFC | Rolandic_Oper_L | -43.5 | | -31.5 | 21 | | 4.4072 | 126 |
| L DLPFC | Frontal_Sup_L | -19.5 | | 10.5 | 49.5 | | 5.5492 | 2548 |
| L DLPFC | Frontal_Mid_R | 28.5 | | 24 | 45 | | 4.642 | 379 |
| R DLPFC | Frontal_Sup_Orb_L | -25.5 | | 55.5 | -1.5 | | 5.5469 | 1241 |
| R DLPFC | Frontal_Mid_R | 43.5 | | 36 | 19.5 | | 72.7833 | 4899 |
| R DLPFC | Insula_R | 36 | | -13.5 | 10.5 | | 5.4603 | 637 |
| R DLPFC | Frontal_Inf_Tri_L | -48 | | 33 | 21 | | 6.1473 | 861 |
| R DLPFC | Rolandic_Oper_L | -40.5 | | -33 | 24 | | 5.3401 | 244 |
| R DLPFC | Frontal_Mid_R | 27 | | 33 | 39 | | 4.9326 | 148 |
| R DLPFC | Frontal_Sup_L | -18 | | 18 | 46.5 | | 4.927 | 306 |

Abbreviations: L, left; R, right; EC, entorhinal cortex; PCC, posterior cingulate cortex; DLPFC, dorsolateral prefrontal cortex; FIC, frontoinsular cortex; DMN, default mode network; SN, salience network; ECN, executive control network; SCD, subjective cognitive decline.

**Supplementary Table 3.** Structural covariance networks seeding from DMN, SN, ECN and their contralateral regions in aMCI group.

| seed | AAL region | MNI coordinates | | | | Peak intensity | Extent |
| --- | --- | --- | --- | --- | --- | --- | --- |
|  |  | X | Y | | Z |  |  |
| L EC | ParaHippocampal_L | -25.5 | | -9 | -28.5 | 77.0739 | 89476 |
| L EC | Frontal_Inf_Orb_L | -30 | | 39 | -18 | 3.7538 | 216 |
| L EC | Frontal_Med_Orb_R | 16.5 | | 55.5 | -4.5 | 4.0633 | 1036 |
| L EC | Lingual_L | -19.5 | | -55.5 | -10.5 | 3.472 | 102 |
| L EC | Frontal_Mid_R | 36 | | 43.5 | 15 | 3.8266 | 267 |
| L EC | SupraMarginal_L | -58.5 | | -22.5 | 40.5 | 4.477 | 2373 |
| R EC | ParaHippocampal_R | 24 | | -9 | -28.5 | 75.1935 | 59996 |
| R EC | Occipital_Mid_L | -33 | | -70.5 | 28.5 | 4.3768 | 1509 |
| R EC | Frontal_Inf_Tri_L | -43.5 | | 36 | 7.5 | 4.3999 | 584 |
| R EC | Frontal_Sup_Medial_R | 7.5 | | 49.5 | 7.5 | 3.6106 | 174 |
| R EC | Cingulum_Mid_R | 4.5 | | -15 | 37.5 | 6.1712 | 12215 |
| R EC | Frontal_Sup_Medial_R | 13.5 | | 54 | 40.5 | 4.2803 | 108 |
| R EC | Frontal_Mid_R | 36 | | 49.5 | 25.5 | 4.3568 | 632 |
| R EC | SupraMarginal_R | 49.5 | | -22.5 | 30 | 3.6898 | 340 |
| R EC | Postcentral_L | -42 | | -30 | 49.5 | 6.0445 | 3338 |
| R EC | Precuneus_L | -12 | | -57 | 67.5 | 4.2897 | 419 |
| L PCC | Cingulum_Mid_L | -1.5 | | -36 | 34.5 | 90.4656 | 211731 |
| L PCC | Thalamus_L | -6 | | -13.5 | 18 | 3.4205 | 156 |
| L PCC | Frontal_Inf_Oper_R | 42 | | 15 | 30 | 4.3271 | 343 |
| L PCC | Frontal_Sup_R | 28.5 | | 28.5 | 51 | 5.0955 | 972 |
| L PCC | Frontal_Mid_R | 28.5 | | 1.5 | 52.5 | 4.5693 | 812 |
| L PCC | Frontal_Sup_L | -13.5 | | 7.5 | 60 | 3.7023 | 217 |
| R PCC | Cingulum_Mid_R | 2 | | -36 | 35 | 91.6029 | 222633 |
| R PCC | Frontal_Mid_R | 28.5 | | 1.5 | 52.5 | 4.287 | 557 |
| R PCC | Precentral_L | -27 | | -9 | 57 | 3.5353 | 156 |
| R PCC | Frontal_Sup_L | -13.5 | | 7.5 | 60 | 3.731 | 275 |
| L FIC | Frontal_Inf_Orb_L | -39 | | 25.5 | -10.5 | 41.7559 | 144662 |
| L FIC | Precentral_L | -40.5 | | 3 | 45 | 4.8494 | 1939 |
| L FIC | Frontal_Mid_R | 30 | | 1.5 | 52.5 | 5.1151 | 1124 |
| L FIC | Parietal_Sup_R | 37.5 | | -49.5 | 63 | 3.328 | 137 |
| R FIC | Frontal_Inf_Orb_R | 39 | | 25.5 | -10.5 | 50.9554 | 228151 |
| R FIC | Frontal_Inf_Oper_R | 45 | | 16.5 | 33 | 3.6723 | 614 |
| R FIC | Precentral_L | -40.5 | | 3 | 46.5 | 3.4937 | 119 |
| L DLPFC | Frontal_Inf_Tri_L | -43.5 | | 36 | 19.5 | 56.6752 | 18194 |
| L DLPFC | Frontal_Sup_Orb_R | 10.5 | | 60 | -15 | 4.7542 | 979 |
| L DLPFC | Temporal_Mid_L | -55.5 | | -60 | 4.5 | 5.0597 | 2771 |
| L DLPFC | Frontal_Inf_Tri_R | 51 | | 43.5 | 4.5 | 4.6963 | 580 |
| L DLPFC | Precuneus_L | -4.5 | | -64.5 | 61.5 | 6.1815 | 4361 |
| L DLPFC | Cingulum_Mid_L | -3 | | -40.5 | 40.5 | 5.1448 | 1641 |
| L DLPFC | Cingulum_Mid_L | -3 | | 25.5 | 34.5 | 4.3623 | 268 |
| L DLPFC | Occipital_Sup_R | 34.5 | | -69 | 40.5 | 5.9181 | 1295 |
| L DLPFC | SupraMarginal_R | 60 | | -40.5 | 34.5 | 3.9983 | 123 |
| L DLPFC | Frontal_Mid_R | 34.5 | | -6 | 52.5 | 4.4196 | 342 |
| R DLPFC | Fusiform_R | 42 | | -37.5 | -15 | 4.804 | 723 |
| R DLPFC | Frontal_Mid_R | 43.5 | | 36 | 19.5 | 59.2965 | 20264 |
| R DLPFC | Frontal_Inf_Tri_L | -40.5 | | 40.5 | 1.5 | 4.9917 | 290 |
| R DLPFC | Frontal_Inf_Oper_L | -52.5 | | 10.5 | 10.5 | 5.9696 | 879 |
| R DLPFC | Occipital_Sup_R | 25.5 | | -75 | 27 | 4.765 | 1374 |
| R DLPFC | Parietal_Inf_L | -33 | | -39 | 43.5 | 4.3997 | 126 |
| R DLPFC | Frontal_Mid_R | 31.5 | | 19.5 | 48 | 4.8265 | 228 |

Abbreviations: L, left; R, right; EC, entorhinal cortex; PCC, posterior cingulate cortex; DLPFC, dorsolateral prefrontal cortex; FIC, frontoinsular cortex; DMN, default mode network; SN, salience network; ECN, executive control network; aMCI, amnestic mild cognitive impairment.

**Supplementary Table 4.** Structural covariance networks seeding from DMN, SN, ECN and their contralateral regions in AD group.

| seed | AAL region | MNI coordinates | | | | | Peak intensity | Extent |
| --- | --- | --- | --- | --- | --- | --- | --- | --- |
|  |  | X | Y | | Z | |  |  |
| L EC | ParaHippocampal_L | -25.5 | | -9 | | -28.5 | 92.3168 | 15623 |
| L EC | Amygdala_R | 30 | | -3 | | -27 | 8.2866 | 14330 |
| L EC | Temporal_Sup_R | 43.5 | | -34.5 | | 6 | 4.8725 | 661 |
| L EC | Angular_R | 37.5 | | -60 | | 33 | 4.2119 | 109 |
| L EC | Occipital_Sup_R | 28.5 | | -75 | | 42 | 4.5367 | 421 |
| L EC | Parietal_Sup_R | 13.5 | | -51 | | 64.5 | 4.1404 | 170 |
| R EC | ParaHippocampal_L | -22.5 | | -9 | | -30 | 8.4161 | 7307 |
| R EC | ParaHippocampal_R | 24 | | -9 | | -28.5 | 85.157 | 25269 |
| R EC | Temporal_Inf_R | 59 | | -54 | | -23 | 5.3482 | 2999 |
| R EC | Occipital_Sup_R | 21 | | -61.5 | | 34.5 | 4.6247 | 224 |
| L PCC | Cingulum_Mid_L | -1.5 | | -36 | | 34.5 | 74.6194 | 85274 |
| L PCC | Lingual_L | -12 | | -83 | | -9 | 3.8396 | 174 |
| L PCC | Frontal_Sup_R | 18 | | 54 | | 28.5 | 3.9007 | 466 |
| R PCC | Cingulum_Mid_R | 1.5 | | -36 | | 36 | 69.5962 | 105053 |
| L FIC | Frontal_Inf_Orb_L | -39 | | 25.5 | | -10.5 | 60.3801 | 147007 |
| L FIC | Lingual_R | 21 | | -82.5 | | -1.5 | 4.0927 | 437 |
| L FIC | Occipital_Mid_L | -40.5 | | -72 | | 19.5 | 3.4409 | 305 |
| L FIC | Calcarine_R | 19.5 | | -66 | | 18 | 3.5507 | 272 |
| R FIC | Frontal_Inf_Orb_R | 37.5 | | 25.5 | | -10.5 | 62.9124 | 118062 |
| R FIC | Fusiform_L | -36 | | -34.5 | | -16.5 | 3.8912 | 335 |
| R FIC | Occipital_Inf_R | 33 | | -82.5 | | -6 | 3.653 | 194 |
| R FIC | Temporal_Sup_L | -54 | | -46.5 | | 18 | 3.4049 | 138 |
| R FIC | Precuneus_R | 12 | | -49.5 | | 19.5 | 3.5819 | 132 |
| L DLPFC | Frontal_Inf_Tri_L | -43.5 | | 36 | | 19.5 | 90.2319 | 130134 |
| L DLPFC | Occipital_Mid_R | 31.5 | | -90 | | 9 | 4.3271 | 645 |
| L DLPFC | Occipital_Sup_L | -16.5 | | -99 | | 15 | 3.9586 | 173 |
| L DLPFC | Cuneus_R | 10.5 | | -84 | | 43.5 | 4.2607 | 208 |
| L DLPFC | Occipital_Sup_L | -16.5 | | -78 | | 45 | 4.8875 | 1179 |
| R DLPFC | Frontal_Mid_R | 43.5 | | 36 | | 19.5 | 63.5045 | 82120 |
| R DLPFC | Precuneus_R | 12 | | -57 | | 37.5 | 5.0245 | 1112 |
| R DLPFC | Occipital_Mid_R | 37.5 | | -64.5 | | 36 | 4.5758 | 529 |
| R DLPFC | Parietal_Inf_R | 28.5 | | -45 | | 51 | 4.3436 | 382 |

Abbreviations: L, left; R, right; EC, entorhinal cortex; PCC, posterior cingulate cortex; DLPFC, dorsolateral prefrontal cortex; FIC, frontoinsular cortex; DMN, default mode network; SN, salience network; ECN, executive control network; AD, Alzheimer’s disease.

**Supplementary Table 5.** Structural covariance networks seeding from anterior and posterior hippocampus in NC group.

| seed | AAL region | MNI coordinates | | | | | Peak intensity | Extent |
| --- | --- | --- | --- | --- | --- | --- | --- | --- |
|  |  | X | Y | | | Z |  |  |
| L aHPC | Hippocampus_L | -24 | | -15 | -19.5 | | 16.5767 | 2078 |
| L aHPC | Cingulum_Ant_L | -3 | | -3 | 31.5 | | 5.2848 | 209 |
| L pHPC | Hippocampus_L | -25.5 | | -31.5 | -4.5 | | 17.2204 | 903 |
| L pHPC | Hippocampus_R | 28.5 | | -31.5 | -7.5 | | 6.7261 | 348 |
| R aHPC | Hippocampus_R | 24 | | -13.5 | -19.5 | | 20.6715 | 1453 |
| R pHPC | Hippocampus_R | 28.5 | | -31.5 | -7.5 | | 15.5482 | 1217 |

Abbreviations: L, left; R, right; aHPC, anterior hippocampus; pHPC, posterior hippocampus; NC, normal control.

**Supplementary Table 6.** Structural covariance networks seeding from anterior and posterior hippocampus in SCD group.

| seed | AAL region | MNI coordinates | | | Peak intensity | Extent |
| --- | --- | --- | --- | --- | --- | --- |
|  |  | X | Y | Z |  |  |
| L aHPC | Hippocampus_L | -25.5 | -13.5 | -18 | 14.0815 | 613 |
| L pHPC | Hippocampus_L | -25.5 | -31.5 | -6 | 13.135 | 623 |
| R aHPC | Hippocampus_R | 25.5 | -13.5 | -19.5 | 18.1889 | 1183 |
| R aHPC | Hippocampus_L | -19.5 | -10.5 | -21 | 6.8778 | 118 |
| R pHPC | Hippocampus_R | 27 | -31.5 | -4.5 | 13.3125 | 588 |
| R pHPC | ParaHippocampal_L | -28.5 | -30 | -12 | 7.0999 | 140 |

Abbreviations: L, left; R, right; aHPC, anterior hippocampus; pHPC, posterior hippocampus; SCD, subjective cognitive decline.

**Supplementary Table 7.** Structural covariance networks seeding from anterior and posterior hippocampus in aMCI group.

| seed | AAL region | MNI coordinates | | | Peak intensity | Extent |
| --- | --- | --- | --- | --- | --- | --- |
|  |  | X | Y | Z |  |  |
| L aHPC | Temporal_Inf_L | -52.5 | -16.5 | -27 | 5.3353 | 489 |
| L aHPC | Hippocampus_L | -24 | -12 | -18 | 34.5451 | 29771 |
| L aHPC | Temporal_Inf_R | 51 | -46.5 | -22.5 | 4.728 | 424 |
| L aHPC | Temporal_Mid_L | -54 | -27 | -12 | 6.3365 | 1266 |
| L aHPC | Fusiform_R | 30 | -79.5 | -15 | 4.512 | 159 |
| L aHPC | Frontal_Inf_Tri_L | -42 | 40.5 | 9 | 5.2233 | 290 |
| L aHPC | Frontal_Mid_R | 33 | 48 | 24 | 4.7186 | 134 |
| L aHPC | Cingulum_Ant_L | -4.5 | 27 | 30 | 4.5207 | 290 |
| L aHPC | Angular_R | 45 | -64.5 | 40.5 | 4.7716 | 532 |
| L aHPC | Temporal_Mid_L | -40.5 | -55.5 | 15 | 5.0459 | 202 |
| L aHPC | Occipital_Sup_L | -18 | -66 | 39 | 5.4854 | 895 |
| L aHPC | Angular_L | -48 | -64.5 | 28.5 | 5.2032 | 337 |
| L aHPC | Angular_L | -54 | -55.5 | 28.5 | 4.2691 | 170 |
| L aHPC | Occipital_Mid_R | 33 | -70.5 | 37.5 | 4.1301 | 202 |
| L aHPC | Postcentral_L | -39 | -33 | 43.5 | 4.9676 | 317 |
| L aHPC | Precuneus_L | 0 | -58.5 | 30 | 6.5179 | 3898 |
| L aHPC | SupraMarginal_R | 40.5 | -31.5 | 40.5 | 4.0187 | 105 |
| L aHPC | Parietal_Sup_L | -21 | -61.5 | 66 | 5.9344 | 692 |
| L pHPC | Temporal_Inf_R | 49.5 | -58.5 | -18 | 4.963 | 431 |
| L pHPC | Fusiform_R | 27 | -78 | -15 | 4.5997 | 230 |
| L pHPC | Frontal_Mid_Orb_R | 37.5 | 46.5 | -12 | 4.3639 | 102 |
| L pHPC | Occipital_Mid_L | -37.5 | -82.5 | 1.5 | 4.7619 | 163 |
| L pHPC | Postcentral_L | -39 | -31.5 | 43.5 | 6.6165 | 1740 |
| L pHPC | Frontal_Inf_Tri_L | -43.5 | 45 | 4.5 | 6.3031 | 323 |
| L pHPC | Occipital_Mid_R | 37.5 | -87 | 12 | 4.8014 | 113 |
| L pHPC | Occipital_Mid_L | -39 | -88.5 | 10.5 | 4.4328 | 108 |
| L pHPC | Precuneus_L | 1.5 | -57 | 30 | 6.0346 | 2961 |
| L pHPC | Postcentral_L | -60 | 0 | 15 | 4.5524 | 102 |
| L pHPC | Cingulum_Mid_L | -9 | 27 | 33 | 5.2903 | 314 |
| L pHPC | Occipital_Sup_L | -19.5 | -63 | 33 | 4.7997 | 654 |
| L pHPC | Occipital_Sup_R | 30 | -72 | 46.5 | 5.5276 | 841 |
| L pHPC | SupraMarginal_R | 60 | -42 | 36 | 4.3882 | 223 |
| L pHPC | Cingulum_Mid_R | 1.5 | -9 | 42 | 4.5444 | 294 |
| L pHPC | Precentral_R | 40.5 | -3 | 51 | 5.6134 | 542 |
| L pHPC | Postcentral_R | 31.5 | -39 | 61.5 | 4.5884 | 170 |
| L pHPC | Parietal_Sup_L | -24 | -61.5 | 64.5 | 6.2087 | 1050 |
| R aHPC | Hippocampus_R | 25.5 | -12 | -18 | 27.0428 | 14784 |
| R aHPC | Temporal_Pole_Sup_L | -46.5 | 21 | -21 | 4.3725 | 108 |
| R aHPC | Fusiform_R | 42 | -52.5 | -18 | 5.0532 | 255 |
| R aHPC | Temporal_Mid_R | 63 | -45 | 4.5 | 6.6178 | 1704 |
| R aHPC | Insula_R | 34.5 | 13.5 | 4.5 | 5.574 | 196 |
| R aHPC | Frontal_Inf_Tri_L | -42 | 39 | 9 | 4.7309 | 164 |
| R aHPC | Calcarine_R | 3 | -58.5 | 15 | 5.2539 | 551 |
| R aHPC | Angular_R | 55.5 | -58.5 | 36 | 4.8037 | 175 |
| R pHPC | Temporal_Mid_L | -61.5 | -46.5 | -3 | 5.6639 | 1758 |
| R pHPC | Hippocampus_R | 27 | -33 | -4.5 | 30.0085 | 20751 |
| R pHPC | Hippocampus_L | -24 | -33 | -1.5 | 11.1101 | 6980 |
| R pHPC | Frontal_Mid_Orb_R | 37.5 | 45 | -13.5 | 4.7613 | 150 |
| R pHPC | Insula_L | -40.5 | 15 | 6 | 6.5086 | 847 |
| R pHPC | Occipital_Mid_R | 39 | -87 | 10.5 | 4.877 | 305 |
| R pHPC | Temporal_Sup_L | -55.5 | -15 | 7.5 | 5.2618 | 701 |
| R pHPC | Frontal_Sup_Medial_L | -9 | 61.5 | 3 | 5.0324 | 126 |
| R pHPC | Frontal_Inf_Tri_L | -43.5 | 42 | 4.5 | 6.6814 | 238 |
| R pHPC | Frontal_Sup_Medial_L | -10.5 | 51 | 16.5 | 4.776 | 147 |
| R pHPC | Precuneus_R | 4.5 | -54 | 19.5 | 6.4258 | 2219 |
| R pHPC | Cuneus_R | 16.5 | -76.5 | 45 | 5.2575 | 698 |
| R pHPC | SupraMarginal_R | 52.5 | -22.5 | 31.5 | 5.5794 | 269 |
| R pHPC | Parietal_Inf_L | -55.5 | -22.5 | 42 | 5.1747 | 611 |
| R pHPC | Precuneus_L | -13.5 | -52.5 | 67.5 | 5.0429 | 350 |

Abbreviations: L, left; R, right; aHPC, anterior hippocampus; pHPC, posterior hippocampus; aMCI, amnestic mild cognitive impairment.

**Supplementary Table 8.** Structural covariance networks seeding from anterior and posterior hippocampus in AD group.

| seed | AAL region | MNI coordinates | | | Peak intensity | Extent |
| --- | --- | --- | --- | --- | --- | --- |
|  |  | X | Y | Z |  |  |
| L aHPC | Hippocampus_L | -22.5 | -13.5 | -18 | 23.6909 | 6166 |
| L aHPC | ParaHippocampal_R | 24 | -18 | -19.5 | 9.2331 | 3486 |
| L pHPC | Temporal_Mid_L | -52.5 | -64.5 | 10.5 | 5.9249 | 153 |
| L pHPC | Angular_L | -51 | -70.5 | 28.5 | 6.6419 | 211 |
| L pHPC | Cingulum_Mid_L | 0 | -37.5 | 37.5 | 5.8268 | 543 |
| L pHPC | Parietal_Sup_L | -24 | -75 | 45 | 4.9698 | 169 |
| L pHPC | Occipital_Mid_R | 39 | -73.5 | 37.5 | 5.4921 | 277 |
| L pHPC | Precuneus_R | 9 | -51 | 67.5 | 5.4694 | 184 |
| R aHPC | Temporal_Inf_R | 52.5 | -7.5 | -37.5 | 6.7455 | 930 |
| R aHPC | ParaHippocampal_R | 22.5 | -12 | -21 | 26.0873 | 7882 |
| R aHPC | Hippocampus_L | -24 | -13.5 | -16.5 | 7.5801 | 2739 |
| R aHPC | Temporal_Mid_R | 58.5 | -58.5 | -1.5 | 5.8812 | 335 |
| R pHPC | Hippocampus_L | -16.5 | -34.5 | 1.5 | 7.525 | 1468 |
| R pHPC | Hippocampus_R | 28.5 | -31.5 | -7.5 | 27.3244 | 3387 |
| R pHPC | Temporal_Inf_R | 55.5 | -30 | -16.5 | 6.2248 | 126 |
| R pHPC | Temporal_Mid_R | 58.5 | -60 | -1.5 | 5.3284 | 111 |

Abbreviations: L, left; R, right; aHPC, anterior hippocampus; pHPC, posterior hippocampus; AD, Alzheimer’s disease.

**Supplementary Table 9.** Structural covariance networks seeding from subregions of the cholinergic basal forebrain in NC group.

| seed | AAL region | MNI coordinates | | | Peak intensity | Extent |
| --- | --- | --- | --- | --- | --- | --- |
|  |  | X | Y | Z |  |  |
| Ch4p | Amygdala_R | 22.5 | -3 | -12 | 15.7728 | 1796 |
| Ch4p | Hippocampus_L | -21 | -10.5 | -18 | 13.5795 | 1716 |
| Ch3 | Temporal_Pole_Sup_R | 37.5 | 13.5 | -25.5 | 5.833 | 137 |
| Ch4al/NSP | Amygdala_R | 19.5 | 3 | -12 | 10.7006 | 770 |
| Ch4al/NSP | Olfactory_L | -16.5 | 7.5 | -13.5 | 10.5352 | 1432 |
| interstitial nuclei | Olfactory_L | -13.5 | 7.5 | -13.5 | 8.7021 | 1230 |
| Ch1/2 | NONE | 1.5 | 7.5 | -6 | 24.4252 | 1477 |

Abbreviations: NSP, Nucleus subputaminalis; NC, normal control.

**Supplementary Table 10.** Structural covariance networks seeding from subregions of the cholinergic basal forebrain in SCD group.

| seed | AAL region | MNI coordinates | | | Peak intensity | Extent |
| --- | --- | --- | --- | --- | --- | --- |
|  |  | X | Y | Z |  |  |
| Ch4p | Amygdala_R | 21 | -3 | -12 | 10.0115 | 1034 |
| Ch4p | Amygdala_L | -22.5 | -4.5 | -18 | 7.4018 | 352 |
| Ch4a-i | Amygdala_L | -18 | 0 | -13.5 | 7.3265 | 117 |
| Ch4al/NSP | Olfactory_L | -21 | 4.5 | -12 | 13.8102 | 526 |
| Ch1/2 | Thalamus_R | 4.5 | -13.5 | 4.5 | 6.3554 | 100 |
| Ch1/2 | Caudate_R | 3 | 9 | -4.5 | 21.9289 | 1077 |

Abbreviations: NSP, Nucleus subputaminalis; SCD, subjective cognitive decline.

**Supplementary Table 11.** Structural covariance networks seeding from subregions of the cholinergic basal forebrain in aMCI group.

| seed | AAL region | MNI coordinates | | | | Peak intensity | Extent |
| --- | --- | --- | --- | --- | --- | --- | --- |
|  |  | X | Y | | Z |  |  |
| Ch4p | Amygdala_R | 24 | | -4.5 | -13.5 | 28.6555 | 15018 |
| Ch4p | Temporal_Inf_R | 49.5 | | -15 | -27 | 4.8512 | 133 |
| Ch4p | Temporal_Inf_R | 49.5 | | -58.5 | -18 | 5.6883 | 517 |
| Ch4p | Temporal_Pole_Sup_L | -45 | | 19.5 | -21 | 4.3467 | 115 |
| Ch4p | Temporal_Inf_R | 63 | | -57 | -3 | 6.1171 | 2934 |
| Ch4p | Frontal_Inf_Orb_R | 43.5 | | 27 | -12 | 5.2372 | 201 |
| Ch4p | Fusiform_R | 28.5 | | -79.5 | -15 | 4.9089 | 103 |
| Ch4p | Temporal_Mid_L | -52.5 | | -27 | -12 | 4.9063 | 339 |
| Ch4p | Occipital_Mid_R | 40.5 | | -85.5 | 3 | 5.4647 | 160 |
| Ch4p | Frontal_Inf_Tri_L | -39 | | 39 | 12 | 4.5282 | 136 |
| Ch4p | Cingulum_Mid_R | 1.5 | | -30 | 36 | 5.423 | 1296 |
| Ch4p | Frontal_Mid_R | 33 | | 46.5 | 21 | 5.0629 | 101 |
| Ch4p | Occipital_Sup_L | -22.5 | | -87 | 22.5 | 4.6002 | 132 |
| Ch4p | Parietal_Inf_R | 51 | | -55.5 | 42 | 5.6745 | 372 |
| Ch4p | Parietal_Inf_L | -48 | | -28.5 | 42 | 4.828 | 399 |
| Ch4p | Precuneus_L | -12 | | -58.5 | 63 | 6.1838 | 420 |
| Ch4a-i | Temporal_Inf_R | 49.5 | | -52.5 | -22.5 | 6.0165 | 574 |
| Ch4a-i | Temporal_Pole_Sup_L | -46.5 | | 21 | -21 | 4.394 | 159 |
| Ch4a-i | Amygdala_L | -13.5 | | 0 | -15 | 13.616 | 18575 |
| Ch4a-i | Temporal_Mid_R | 67.5 | | -46.5 | 1.5 | 5.3524 | 2316 |
| Ch4a-i | Fusiform_R | 27 | | -79.5 | -15 | 4.9542 | 131 |
| Ch4a-i | Temporal_Mid_L | -52.5 | | -27 | -12 | 6.459 | 1102 |
| Ch4a-i | Frontal_Sup_Orb_R | 18 | | 55.5 | -4.5 | 4.832 | 105 |
| Ch4a-i | Temporal_Mid_L | -57 | | -52.5 | 10.5 | 4.7452 | 261 |
| Ch4a-i | Occipital_Sup_L | -22.5 | | -87 | 22.5 | 5.2639 | 311 |
| Ch4a-i | Cingulum_Post_L | 0 | | -34.5 | 28.5 | 4.2215 | 154 |
| Ch4a-i | Occipital_Mid_L | -31.5 | | -70.5 | 39 | 4.447 | 180 |
| Ch4a-i | SupraMarginal_R | 52.5 | | -24 | 33 | 4.7247 | 156 |
| Ch4a-i | Postcentral_L | -37.5 | | -28.5 | 45 | 5.8399 | 532 |
| Ch4a-i | SupraMarginal_R | 39 | | -30 | 42 | 5.4964 | 493 |
| Ch4a-i | Precuneus_L | -12 | | -58.5 | 63 | 7.3207 | 649 |
| Ch4a-i | Postcentral_R | 21 | | -39 | 61.5 | 4.8356 | 197 |
| Ch3 | Temporal_Inf_R | 49.5 | | -52.5 | -22.5 | 7.003 | 734 |
| Ch3 | Temporal_Pole_Sup_L | -46.5 | | 21 | -21 | 4.4424 | 243 |
| Ch3 | Temporal_Inf_L | -54 | | -42 | -18 | 5.0149 | 206 |
| Ch3 | Fusiform_R | 27 | | -79.5 | -15 | 4.9626 | 125 |
| Ch3 | Temporal_Mid_L | -52.5 | | -27 | -12 | 6.0224 | 1553 |
| Ch3 | Frontal_Med_Orb_R | 9 | | 61.5 | -4.5 | 4.2415 | 112 |
| Ch3 | Temporal_Inf_R | 61.5 | | -46.5 | -12 | 5.4179 | 2169 |
| Ch3 | Frontal_Sup_Medial_R | 7.5 | | 54 | 16.5 | 4.595 | 147 |
| Ch3 | Occipital_Sup_L | -22.5 | | -87 | 22.5 | 4.462 | 157 |
| Ch3 | Cingulum_Post_L | 0 | | -34.5 | 28.5 | 4.4764 | 343 |
| Ch3 | Occipital_Mid_L | -27 | | -72 | 31.5 | 4.6987 | 214 |
| Ch3 | Postcentral_L | -39 | | -31.5 | 45 | 5.5666 | 477 |
| Ch3 | SupraMarginal_R | 39 | | -31.5 | 42 | 4.5929 | 233 |
| Ch3 | Postcentral_R | 21 | | -39 | 61.5 | 5.4482 | 257 |
| Ch3 | Precuneus_L | -12 | | -58.5 | 63 | 6.169 | 469 |
| Ch4al/NSP | Temporal_Inf_R | 49.5 | | -52.5 | -22.5 | 6.0914 | 437 |
| Ch4al/NSP | Temporal_Mid_L | -56 | | -54 | 12 | 5.2369 | 757 |
| Ch4al/NSP | Temporal_Mid_R | 57 | | -48 | 9 | 5.3407 | 1751 |
| Ch4al/NSP | Temporal_Mid_R | 48 | | 1.5 | -16.5 | 4.5441 | 121 |
| Ch4al/NSP | Temporal_Mid_L | -63 | | -34.5 | -3 | 5.5881 | 1557 |
| Ch4al/NSP | Occipital_Mid_L | -40.5 | | -82.5 | 1.5 | 4.9098 | 152 |
| Ch4al/NSP | Occipital_Sup_L | -21 | | -85.5 | 25.5 | 4.6792 | 100 |
| Ch4al/NSP | Cingulum_Mid_L | -9 | | 27 | 33 | 5.113 | 142 |
| Ch4al/NSP | Postcentral_L | -36 | | -28.5 | 46.5 | 5.2439 | 288 |
| Ch4al/NSP | SupraMarginal_R | 39 | | -31.5 | 43.5 | 4.4024 | 197 |
| Ch4al/NSP | Frontal_Mid_L | -30 | | 24 | 43.5 | 4.8451 | 136 |
| Ch4al/NSP | Postcentral_R | 19.5 | | -40.5 | 61.5 | 5.5355 | 300 |
| Ch4al/NSP | Parietal_Sup_L | -18 | | -42 | 63 | 5.2381 | 165 |
| interstitial nuclei | Hippocampus_L | -30 | | -15 | -10.5 | 5.7511 | 363 |
| interstitial nuclei | Insula_R | 33 | | 22.5 | -4.5 | 4.7017 | 103 |
| interstitial nuclei | Frontal_Sup_Orb_R | 18 | | 55.5 | -4.5 | 4.9143 | 161 |
| interstitial nuclei | Postcentral_R | 37.5 | | -30 | 43.5 | 4.9291 | 238 |
| interstitial nuclei | Precuneus_L | -12 | | -60 | 63 | 6.9954 | 182 |
| Ch1/2 | Temporal_Inf_L | -55.5 | | -15 | -28.5 | 5.3101 | 748 |
| Ch1/2 | Caudate_R | 3 | | 7.5 | -6 | 33.8704 | 37761 |
| Ch1/2 | Frontal_Mid_Orb_L | -27 | | 33 | -16.5 | 5.6778 | 134 |
| Ch1/2 | Lingual_L | -16.5 | | -79.5 | -13.5 | 5.3801 | 240 |
| Ch1/2 | Frontal_Inf_Orb_R | 39 | | 39 | -12 | 5.0893 | 121 |
| Ch1/2 | Temporal_Inf_L | -54 | | -42 | -18 | 6.4365 | 1437 |
| Ch1/2 | Temporal_Mid_L | -57 | | -51 | 10.5 | 5.0455 | 292 |
| Ch1/2 | Temporal_Mid_R | 49.5 | | -48 | 9 | 4.9765 | 176 |
| Ch1/2 | Thalamus_R | 18 | | -15 | 15 | 4.3868 | 132 |
| Ch1/2 | Rolandic_Oper_R | 54 | | -12 | 12 | 4.9252 | 701 |
| Ch1/2 | Thalamus_L | -4.5 | | -10.5 | 13.5 | 4.209 | 113 |
| Ch1/2 | Temporal_Mid_L | -42 | | -66 | 13.5 | 5.3746 | 127 |
| Ch1/2 | Angular_R | 52.5 | | -63 | 31.5 | 5.3422 | 575 |
| Ch1/2 | Occipital_Mid_L | -28.5 | | -72 | 30 | 4.9499 | 410 |
| Ch1/2 | Angular_L | -46.5 | | -69 | 28.5 | 4.0442 | 149 |
| Ch1/2 | Precuneus_L | 0 | | -58.5 | 30 | 4.3989 | 126 |
| Ch1/2 | Cingulum_Mid_R | 6 | | -10.5 | 40.5 | 6.2382 | 932 |
| Ch1/2 | Postcentral_R | 46.5 | | -24 | 42 | 4.1402 | 357 |
| Ch1/2 | Cingulum_Mid_L | -9 | | -10.5 | 45 | 4.1981 | 114 |
| Ch1/2 | Postcentral_R | 25.5 | | -37.5 | 60 | 4.8179 | 185 |
| Ch1/2 | Precuneus_L | -12 | | -60 | 63 | 5.8125 | 210 |

Abbreviations: NSP, Nucleus subputaminalis; aMCI, amnestic mild cognitive impairment.

**Supplementary Table 12.** Structural covariance networks seeding from subregions of the cholinergic basal forebrain in AD group.

| seed | AAL region | MNI coordinates | | | | Peak intensity | Extent |
| --- | --- | --- | --- | --- | --- | --- | --- |
|  |  | X | Y | | Z |  |  |
| Ch4p | Amygdala_R | 24 | | -4.5 | -13.5 | 22.8623 | 10398 |
| Ch4p | Amygdala_L | -24 | | -3 | -13.5 | 9.79 | 3253 |
| Ch4p | Temporal_Inf_R | 58.5 | | -63 | -4.5 | 6.2344 | 536 |
| Ch4a-i | Fusiform_R | 34.5 | | -4.5 | -43.5 | 5.1988 | 147 |
| Ch4a-i | Temporal_Mid_R | 61.5 | | -54 | 4.5 | 6.6461 | 1369 |
| Ch4a-i | Temporal_Mid_R | 66 | | -31.5 | -1.5 | 4.9744 | 135 |
| Ch3 | Amygdala_R | 19.5 | | 0 | -12 | 14.8614 | 15254 |
| Ch3 | Temporal_Inf_R | 55.5 | | -66 | -6 | 7.172 | 1947 |
| Ch4al/NSP | Temporal_Mid_R | 60 | | -54 | 4.5 | 7.0444 | 3140 |
| Ch4al/NSP | Amygdala_R | 23 | | 3 | -10.5 | 14.3931 | 7970 |
| Ch4al/NSP | Frontal_Mid_Orb_L | -22.5 | | 39 | -18 | 5.2284 | 244 |
| Ch4al/NSP | Occipital_Inf_R | 37.5 | | -85.5 | -12 | 5.7338 | 410 |
| Ch4al/NSP | Frontal_Inf_Orb_R | 46.5 | | 25.5 | -7.5 | 5.3194 | 246 |
| interstitial nuclei | Temporal_Inf_R | 60 | | -25.5 | -22.5 | 7.072 | 1121 |
| interstitial nuclei | Temporal_Mid_R | 54 | | -66 | 3 | 7.2451 | 1299 |
| interstitial nuclei | Precentral_R | 61.5 | | 12 | 16.5 | 4.9672 | 155 |
| Ch1/2 | Caudate_R | 3 | | 7.5 | -4.5 | 28.7392 | 20469 |
| Ch1/2 | Temporal_Inf_L | -37.5 | | -25.5 | -22.5 | 5.4576 | 293 |
| Ch1/2 | Temporal_Inf_R | 58.5 | | -49.5 | -15 | 7.1628 | 3972 |
| Ch1/2 | Temporal_Inf_L | -48 | | -49.5 | -13.5 | 5.0212 | 329 |
| Ch1/2 | Frontal_Sup_Orb_R | 15 | | 61.5 | -6 | 4.9001 | 367 |
| Ch1/2 | Heschl_R | 51 | | -7.5 | 6 | 4.7717 | 100 |
| Ch1/2 | Frontal_Sup_Medial_L | -12 | | 63 | 18 | 5.2455 | 240 |
| Ch1/2 | Temporal_Mid_L | -43.5 | | -54 | 15 | 6.0274 | 185 |
| Ch1/2 | Temporal_Sup_L | -61.5 | | -45 | 16.5 | 4.4755 | 102 |
| Ch1/2 | Frontal_Inf_Oper_R | 36 | | 13.5 | 31.5 | 4.6925 | 149 |
| Ch1/2 | Frontal_Mid_R | 25.5 | | 48 | 33 | 5.7443 | 113 |
| Ch1/2 | Cingulum_Mid_R | 7.5 | | 12 | 36 | 5.3086 | 345 |

Abbreviations: NSP, Nucleus subputaminalis; AD, Alzheimer’s disease.

**Supplementary Table 13.** Significant between-group(NC-SCD) differences in structural covariance networks seeding from DMN, SN, ECN and their contralateral regions.

| seed | Peak region (AAL) | MNI coordinates | | | | Peak intensity | Extent |
| --- | --- | --- | --- | --- | --- | --- | --- |
|  |  | X | Y | | Z |  |  |
| L EC | Temporal_Mid_L | -69 | | -24 | 3 | -3.4565 | 376 |
| R EC | Temporal_Inf_L | -49.5 | | -36 | -18 | -2.9509 | 122 |
| R EC | Temporal_Mid_L | -70.5 | | -30 | -1.5 | -3.2285 | 172 |
| R EC | SupraMarginal_L | -60 | | -40.5 | 31.5 | -2.889 | 153 |
| R EC | SupraMarginal_R | 63 | | -24 | 43.5 | -3.9511 | 1199 |
| R EC | Parietal_Sup_L | -19.5 | | -55.5 | 60 | -3.2705 | 435 |
| R EC | Parietal_Sup_R | 15 | | -73.5 | 60 | -3.2041 | 199 |
| L PCC | Temporal_Inf_R | 49.5 | | -36 | -16.5 | -4.2714 | 2273 |
| L PCC | Calcarine_R | 25.5 | | -46.5 | 4.5 | -3.0543 | 188 |
| L PCC | Temporal_Mid_L | -54 | | -55.5 | 6 | -3.0803 | 272 |
| L PCC | Frontal_Inf_Tri_R | 45 | | 37.5 | 10.5 | -2.8461 | 149 |
| L PCC | Occipital_Mid_L | -36 | | -88.5 | 10.5 | -3.1437 | 228 |
| L PCC | Occipital_Sup_L | -27 | | -93 | 22.5 | -3.7092 | 261 |
| L PCC | Temporal_Sup_R | 57 | | -27 | 12 | -2.9576 | 104 |
| L PCC | Occipital_Sup_R | 15 | | -88.5 | 30 | -3.9183 | 616 |
| L PCC | Frontal_Mid_R | 28.5 | | 21 | 39 | -3.3222 | 468 |
| L PCC | Precuneus_R | 3 | | -51 | 45 | 3.2657 | 357 |
| L PCC | Precentral_R | 27 | | -18 | 57 | -3.3307 | 718 |
| R PCC | Temporal_Inf_R | 49.5 | | -36 | -16.5 | -4.4394 | 2438 |
| R PCC | Temporal_Mid_L | -49.5 | | -28.5 | -16.5 | -3.4148 | 274 |
| R PCC | Precuneus_R | 27 | | -46.5 | 3 | -3.1244 | 327 |
| R PCC | Occipital_Mid_L | -34.5 | | -85.5 | 10.5 | -3.3284 | 352 |
| R PCC | Occipital_Sup_L | -27 | | -93 | 22.5 | -3.602 | 187 |
| R PCC | Cuneus_R | 13.5 | | -88.5 | 28.5 | -4.089 | 732 |
| R PCC | Cingulum_Mid_R | 3 | | -4.5 | 37.5 | -3.2127 | 175 |
| R PCC | Paracentral_Lobule_R | 13.5 | | -30 | 60 | -3.4301 | 827 |
| R PCC | Frontal_Mid_R | 30 | | 19.5 | 40.5 | -3.6463 | 1164 |
| R PCC | Precuneus_R | 6 | | -51 | 43.5 | 3.3485 | 273 |
| R PCC | Paracentral_Lobule_L | -16.5 | | -16.5 | 66 | -4.0033 | 2436 |
| L FIC | Temporal_Inf_R | 49.5 | | -42 | -13.5 | -4.0352 | 2675 |
| L FIC | Precuneus_R | 19.5 | | -42 | 9 | -3.5171 | 255 |
| L FIC | Postcentral_R | 66 | | -13.5 | 33 | -4.0219 | 3124 |
| L FIC | Cuneus_L | -3 | | -82.5 | 21 | -3.295 | 498 |
| L FIC | SupraMarginal_L | -57 | | -39 | 30 | -4.4318 | 1627 |
| L FIC | Cingulum_Mid_L | 0 | | -3 | 39 | -2.9844 | 344 |
| L FIC | Frontal_Inf_Oper_R | 43.5 | | 15 | 33 | -3.2982 | 294 |
| L FIC | Postcentral_L | -46.5 | | -9 | 49.5 | -4.0589 | 809 |
| L FIC | Precentral_R | 43.5 | | -3 | 49.5 | -3.7526 | 1281 |
| L FIC | Parietal_Sup_L | -27 | | -61.5 | 61.5 | -2.9357 | 167 |
| L FIC | Frontal_Mid_R | 42 | | 19.5 | 55.5 | -3.1666 | 187 |
| R FIC | Rectus_L | -7.5 | | 21 | -18 | -3.654 | 483 |
| R FIC | Temporal_Mid_R | 63 | | -34.5 | -7.5 | -3.2708 | 713 |
| R FIC | Temporal_Mid_R | 49.5 | | -36 | -13.5 | -3.2485 | 196 |
| R FIC | Parietal_Inf_L | -34.5 | | -45 | 48 | -3.8531 | 897 |
| L DLPFC | Temporal_Sup_R | 64.5 | | -33 | 13.5 | -4.1177 | 3105 |
| L DLPFC | Temporal_Pole_Sup_L | -51 | | 18 | -10.5 | -2.9663 | 133 |
| L DLPFC | Temporal_Mid_L | -66 | | -30 | -4.5 | -3.2682 | 986 |
| L DLPFC | Occipital_Mid_L | -39 | | -70.5 | 3 | 3.1967 | 169 |
| L DLPFC | Precuneus_R | 19.5 | | -45 | 9 | -3.3463 | 155 |
| L DLPFC | SupraMarginal_L | -58.5 | | -40.5 | 33 | -3.7466 | 1190 |
| L DLPFC | Parietal_Sup_L | -28.5 | | -64.5 | 58.5 | -3.2355 | 320 |
| L DLPFC | Postcentral_L | -48 | | -7.5 | 49.5 | -3.0546 | 181 |
| R DLPFC | Temporal_Inf_R | 54 | | -48 | -27 | -3.3482 | 338 |
| R DLPFC | Temporal_Mid_R | 67.5 | | -34.5 | -13.5 | -3.0648 | 325 |
| R DLPFC | Temporal_Mid_L | -64.5 | | -34.5 | -3 | -2.9526 | 202 |
| R DLPFC | Occipital_Mid_L | -39 | | -67.5 | 1.5 | 3.4323 | 313 |

Abbreviations: L, left; R, right; EC, entorhinal cortex; PCC, posterior cingulate cortex; DLPFC, dorsolateral prefrontal cortex; FIC, frontoinsular cortex; DMN, default mode network; SN, salience network; ECN, executive control network; NC, normal control; SCD, subjective cognitive decline.

**Supplementary Table 14.** Significant between-group(HC-aMCI) differences in structural covariance networks seeding from DMN, SN, ECN and their contralateral regions.

| seed | Peak region (AAL) | MNI coordinates | | | Peak intensity | Extent |
| --- | --- | --- | --- | --- | --- | --- |
|  |  | X | Y | Z |  |  |
| L EC | Temporal_Pole_Mid_R | 46.5 | 7.5 | -30 | -2.9287 | 332 |
| L EC | Postcentral_L | -40.5 | -9 | 33 | -3.1842 | 100 |
| L EC | Frontal_Sup_R | 16.5 | 36 | 34.5 | -3.2666 | 347 |
| L EC | Parietal_Sup_L | -22.5 | -61.5 | 55.5 | -3.1814 | 198 |
| L EC | Precentral_R | 22.5 | -21 | 73.5 | -3.7033 | 1538 |
| R EC | Temporal_Mid_R | 52.5 | 6 | -28.5 | -2.8569 | 111 |
| R EC | Rectus_L | -4.5 | 33 | -25.5 | -3.0246 | 246 |
| R EC | Frontal_Mid_Orb_L | -33 | 63 | -6 | -3.1437 | 250 |
| R EC | Cingulum_Mid_L | -9 | 1.5 | 31.5 | -3.4764 | 431 |
| R EC | Frontal_Mid_L | -33 | 46.5 | 19.5 | -3.5458 | 622 |
| R EC | Temporal_Sup_R | 54 | -30 | 19.5 | -3.0386 | 114 |
| R EC | Precentral_L | -37.5 | -7.5 | 39 | -3.9178 | 677 |
| R EC | Parietal_Sup_L | -19.5 | -58.5 | 55.5 | -3.1564 | 159 |
| R EC | Frontal_Sup_R | 13.5 | -1.5 | 76.5 | -3.0921 | 424 |
| L PCC | Frontal_Mid_L | -25.5 | 24 | 34.5 | -5.4367 | 2022 |
| L PCC | Temporal_Mid_R | 51 | -58.5 | 16.5 | 3.3112 | 431 |
| L PCC | SupraMarginal_R | 49.5 | -30 | 30 | -5.0804 | 2419 |
| L PCC | Precentral_L | -40.5 | -4.5 | 30 | -3.7648 | 217 |
| L PCC | Precentral_R | 15 | -24 | 76.5 | -4.5769 | 2876 |
| L PCC | Precentral_L | -46.5 | -7.5 | 54 | -2.9063 | 104 |
| R PCC | Temporal_Mid_R | 51 | -58.5 | 16.5 | 3.235 | 352 |
| R PCC | SupraMarginal_R | 52.5 | -30 | 30 | -4.8576 | 2325 |
| R PCC | Precentral_L | -40.5 | -4.5 | 30 | -3.8388 | 220 |
| R PCC | Precentral_R | 15 | -24 | 76.5 | -4.3856 | 2539 |
| L FIC | Temporal_Mid_R | 49.5 | -46.5 | 6 | 3.2717 | 166 |
| L FIC | SupraMarginal_R | 58.5 | -27 | 19.5 | -3.6792 | 750 |
| L FIC | Precentral_R | 48 | 1.5 | 22.5 | -3.3924 | 311 |
| L FIC | Precentral_R | 52.5 | 0 | 48 | -2.9781 | 150 |
| L FIC | Postcentral_R | 16.5 | -28.5 | 79.5 | -3.5059 | 440 |
| L FIC | Paracentral_Lobule_L | -13.5 | -28.5 | 78 | -3.7093 | 302 |
| R FIC | Temporal_Inf_R | 54 | -16.5 | -28.5 | 3.0038 | 115 |
| R FIC | Temporal_Mid_R | 52.5 | -63 | 15 | 3.4561 | 429 |
| R FIC | Cingulum_Mid_L | -9 | -37.5 | 43.5 | 3.6384 | 2912 |
| R FIC | Parietal_Sup_L | -22.5 | -49.5 | 67.5 | 3.0636 | 190 |
| R FIC | Paracentral_Lobule_L | -13.5 | -28.5 | 73.5 | -3.6751 | 603 |
| R FIC | Precentral_R | 15 | -27 | 79.5 | -4.7806 | 938 |
| L DLPFC | Temporal_Pole_Sup_R | 40.5 | 9 | -24 | -2.8693 | 107 |
| L DLPFC | Temporal_Sup_R | 55.5 | -28.5 | 16.5 | -3.7861 | 1499 |
| L DLPFC | Temporal_Sup_L | -51 | -30 | 6 | -3.3117 | 396 |
| L DLPFC | Frontal_Sup_Medial_R | 16.5 | 48 | 7.5 | -3.2349 | 177 |
| L DLPFC | Precentral_L | -37.5 | -6 | 39 | -3.5684 | 194 |
| L DLPFC | Precuneus_L | -3 | -73.5 | 55.5 | 3.3564 | 853 |
| R DLPFC | Cingulum_Mid_R | 7.5 | -39 | 43.5 | 3.1672 | 269 |

Abbreviations: L, left; R, right; EC, entorhinal cortex; PCC, posterior cingulate cortex; DLPFC, dorsolateral prefrontal cortex; FIC, frontoinsular cortex; DMN, default mode network; SN, salience network; ECN, executive control network; NC, normal control; aMCI, amnestic mild cognitive impairment.

**Supplementary Table 15.** Significant between-group(HC-AD) differences in structural covariance networks seeding from DMN, SN, ECN and their contralateral regions.

| seed | Peak region (AAL) | MNI coordinates | | | | Peak intensity | Extent |
| --- | --- | --- | --- | --- | --- | --- | --- |
|  |  | X | Y | | Z |  |  |
| L EC | Temporal_Mid_L | -67.5 | | -13.5 | -18 | -3.3227 | 284 |
| L EC | Temporal_Mid_L | -57 | | -37.5 | -1.5 | -3.018 | 924 |
| L EC | Thalamus_L | -12 | | -12 | 0 | -4.5871 | 1024 |
| L EC | Temporal_Sup_R | 70.5 | | -28.5 | 6 | -3.1888 | 261 |
| L EC | Frontal_Sup_Medial_L | -7.5 | | 52.5 | 12 | -3.0338 | 454 |
| L EC | Temporal_Sup_R | 37.5 | | -27 | 12 | -3.7091 | 903 |
| L EC | Precuneus_L | -13.5 | | -54 | 37.5 | 4.0069 | 180 |
| L EC | Frontal_Sup_R | 22.5 | | 34.5 | 39 | -3.0588 | 162 |
| L EC | Frontal_Mid_L | -22.5 | | 35 | 39 | -3.714 | 445 |
| L EC | Precuneus_R | 9 | | -64.5 | 67.5 | -3.214 | 210 |
| L EC | Frontal_Sup_R | 15 | | -3 | 76.5 | -3.7485 | 358 |
| R EC | ParaHippocampal_R | 18 | | -1.5 | -22.5 | 3.267 | 273 |
| R EC | Temporal_Mid_L | -54 | | -49.5 | 3 | -3.381 | 1596 |
| R EC | Thalamus_L | -12 | | -10.5 | 0 | -4.111 | 666 |
| R EC | Frontal_Sup_R | 13.5 | | 67.5 | 19.5 | -3.01 | 426 |
| R EC | Precentral_L | -43.5 | | -6 | 43.5 | -4.5547 | 4059 |
| R EC | Temporal_Sup_R | 39 | | -27 | 12 | -3.0484 | 383 |
| R EC | Precentral_R | 54 | | -6 | 49.5 | -3.1886 | 812 |
| R EC | Frontal_Mid_L | -30 | | 28.5 | 36 | -3.6253 | 221 |
| R EC | Parietal_Sup_L | -16.5 | | -60 | 61.5 | -4.414 | 1601 |
| R EC | Postcentral_L | -36 | | -37.5 | 67.5 | -3.3462 | 259 |
| R EC | Frontal_Sup_R | 15 | | -1.5 | 76.5 | -3.841 | 469 |
| L PCC | Precuneus_L | -10.5 | | -49.5 | 10.5 | -3.0941 | 380 |
| L PCC | Temporal_Mid_R | 42 | | -52.5 | 19.5 | 4.0244 | 277 |
| L PCC | Precuneus_L | -3 | | -51 | 55.5 | 3.7516 | 757 |
| L PCC | Precentral_R | 31.5 | | -21 | 70.5 | -3.1332 | 686 |
| L PCC | Precentral_L | -16.5 | | -16.5 | 72 | -3.7043 | 577 |
| L PCC | Frontal_Sup_R | 13.5 | | -6 | 76.5 | -3.182 | 100 |
| R PCC | Precuneus_L | -7.5 | | -48 | 10.5 | -2.9724 | 280 |
| R PCC | Temporal_Sup_R | 67.5 | | -25.5 | 9 | -3.6774 | 615 |
| R PCC | Temporal_Mid_R | 42 | | -52.5 | 19.5 | 3.8111 | 256 |
| R PCC | Precuneus_L | -1.5 | | -49.5 | 57 | 3.6326 | 718 |
| R PCC | Precentral_R | 21 | | -24 | 75 | -2.965 | 454 |
| R PCC | Precentral_L | -16.5 | | -16.5 | 72 | -3.7128 | 631 |
| L FIC | Temporal_Mid_R | 42 | | -49.5 | 18 | 3.4797 | 150 |
| L FIC | SupraMarginal_L | -57 | | -40.5 | 28.5 | -3.9307 | 569 |
| L FIC | Precentral_R | 45 | | -3 | 51 | -4.0778 | 1420 |
| L FIC | Postcentral_L | -49.5 | | -9 | 54 | -3.0581 | 168 |
| L FIC | Parietal_Sup_L | -16.5 | | -63 | 57 | -4.4814 | 945 |
| L FIC | Parietal_Sup_R | 31.5 | | -57 | 66 | -3.4062 | 220 |
| L FIC | Paracentral_Lobule_L | -12 | | -27 | 79.5 | -3.2654 | 205 |
| R FIC | Frontal_Sup_Orb_R | 15 | | 39 | -22.5 | 3.0417 | 193 |
| R FIC | Angular_R | 42 | | -54 | 30 | 3.0122 | 183 |
| R FIC | Postcentral_L | -49.5 | | -9 | 54 | -3.0941 | 169 |
| R FIC | Supp_Motor_Area_R | 12 | | -18 | 49.5 | 3.1579 | 113 |
| R FIC | Precentral_R | 15 | | -27 | 75 | -3.8561 | 393 |
| R FIC | Paracentral_Lobule_L | -18 | | -25.5 | 78 | -2.8967 | 189 |
| L DLPFC | Temporal_Inf_R | 54 | | -36 | -27 | -3.3141 | 616 |
| L DLPFC | Temporal_Inf_L | -46.5 | | -48 | -24 | -3.1515 | 104 |
| L DLPFC | Temporal_Mid_L | -69 | | -12 | -15 | -3.4637 | 1064 |
| L DLPFC | Lingual_L | -9 | | 39 | 3 | -3.9733 | 2265 |
| L DLPFC | Insula_R | 31.5 | | -24 | 16.5 | -4.5449 | 3028 |
| L DLPFC | Parietal_Inf_L | -39 | | -42 | 39 | -3.7146 | 805 |
| L DLPFC | Precuneus_L | -7.5 | | -57 | 13.5 | -3.2549 | 561 |
| L DLPFC | Parietal_Sup_L | -22.5 | | -69 | 57 | -3.9615 | 559 |
| L DLPFC | Precuneus_R | 15 | | -54 | 46.5 | 3.3861 | 372 |
| L DLPFC | Frontal_Sup_L | -15 | | 16.5 | 45 | -3.4276 | 180 |
| L DLPFC | Parietal_Sup_R | 30 | | -55.5 | 59 | -2.9259 | 123 |
| L DLPFC | Supp_Motor_Area_R | 10.5 | | 4.5 | 75 | -3.0956 | 161 |
| R DLPFC | Insula_L | -27 | | 21 | -10.5 | 2.9381 | 300 |
| R DLPFC | Frontal_Sup_L | -27 | | 63 | 7.5 | 3.3359 | 181 |
| R DLPFC | Calcarine_L | -15 | | -49.5 | 10.5 | -3.0684 | 106 |
| R DLPFC | Temporal_Mid_R | 42 | | -51 | 19.5 | 3.5584 | 204 |
| R DLPFC | Precuneus_R | 12 | | -57 | 40.5 | 3.602 | 249 |

Abbreviations: L, left; R, right; EC, entorhinal cortex; PCC, posterior cingulate cortex; DLPFC, dorsolateral prefrontal cortex; FIC, frontoinsular cortex; DMN, default mode network; SN, salience network; ECN, executive control network; NC, normal control; AD, Alzheimer’s disease.

**Supplementary Table 16.** Significant between-group(NC-SCD) differences in structural covariance networks seeding from anterior and posterior hippocampus.

| seed | AAL region | MNI coordinates | | | | Peak intensity | Extent |
| --- | --- | --- | --- | --- | --- | --- | --- |
|  |  | X | Y | | Z |  |  |
| L aHPC | Temporal_Mid_L | -52.5 | | -49.5 | 0 | -3.7864 | 173 |
| L aHPC | Frontal_Sup_Medial_L | 1.5 | | 37.5 | 33 | -3.5833 | 140 |
| L pHPC | Temporal_Inf_R | 52.5 | | -9 | -24 | -3.8517 | 405 |
| L pHPC | Temporal_Inf_L | -49.5 | | -27 | -27 | -3.8777 | 231 |
| L pHPC | Temporal_Mid_R | 58.5 | | -34.5 | -7.5 | -4.3787 | 615 |
| L pHPC | Temporal_Mid_R | 49.5 | | -48 | 15 | -5.1987 | 250 |
| L pHPC | Cingulum_Mid_R | 12 | | -27 | 45 | -3.1324 | 125 |
| L pHPC | Frontal_Sup_R | 27 | | 13.5 | 57 | -4.0136 | 768 |
| L pHPC | Precentral_R | 13.5 | | -28.5 | 75 | -3.9158 | 785 |
| L pHPC | Frontal_Sup_L | -15 | | 22.5 | 63 | -4.2541 | 854 |
| L pHPC | Supp_Motor_Area_L | -12 | | -12 | 64.5 | -4.161 | 171 |
| R aHPC | ParaHippocampal_L | -30 | | -16.5 | -25.5 | -4.1914 | 115 |
| R aHPC | Temporal_Mid_R | 61.5 | | -34.5 | -6 | -4.2278 | 409 |
| R aHPC | Occipital_Mid_L | -42 | | -73.5 | 1.5 | 3.788 | 123 |
| R aHPC | Temporal_Mid_L | -49.5 | | -55.5 | 3 | -3.5433 | 135 |
| R aHPC | SupraMarginal_R | 58.5 | | -28.5 | 42 | -4.3683 | 516 |
| R aHPC | Precuneus_R | 9 | | -42 | 61.5 | -3.4651 | 132 |
| R pHPC | Temporal_Inf_L | -48 | | -42 | -27 | -3.6242 | 135 |
| R pHPC | Temporal_Inf_R | 54 | | -31.5 | -24 | -4.0403 | 115 |
| R pHPC | Temporal_Inf_R | 61.5 | | -40.5 | -19.5 | -4.1468 | 837 |
| R pHPC | Temporal_Mid_R | 49.5 | | -48 | 15 | -3.9347 | 124 |
| R pHPC | Frontal_Sup_L | -19.5 | | 33 | 42 | -3.417 | 203 |
| R pHPC | Cingulum_Mid_R | 12 | | -27 | 45 | -3.2953 | 147 |
| R pHPC | Precentral_R | 45 | | -1.5 | 42 | -3.4563 | 113 |
| R pHPC | Precentral_R | 33 | | -16.5 | 51 | -3.2985 | 107 |
| R pHPC | Frontal_Sup_L | -16.5 | | 22.5 | 63 | -4.5136 | 622 |
| R pHPC | Precentral_R | 13.5 | | -28.5 | 75 | -3.9334 | 412 |

Abbreviations: L, left; R, right; aHPC, anterior hippocampus; pHPC, posterior hippocampus; NC, normal control; SCD, subjective cognitive decline.

**Supplementary Table 17.** Significant between-group(NC-aMCI) differences in structural covariance networks seeding from anterior and posterior hippocampus.

| seed | AAL region | MNI coordinates | | | | Peak intensity | Extent |
| --- | --- | --- | --- | --- | --- | --- | --- |
|  |  | X | Y | | Z |  |  |
| L aHPC | Temporal_Inf_L | -43.5 | | 7.5 | -31.5 | -3.8729 | 162 |
| L aHPC | Hippocampus_L | -25.5 | | -13.5 | -13.5 | 5.0774 | 239 |
| L aHPC | Temporal_Sup_R | 43.5 | | -27 | 10.5 | -3.6815 | 172 |
| L aHPC | Temporal_Sup_R | 55.5 | | -30 | 16.5 | -3.9659 | 265 |
| L aHPC | Cingulum_Mid_L | -6 | | -1.5 | 34.5 | -3.7299 | 172 |
| L aHPC | Frontal_Sup_Medial_L | 1.5 | | 46.5 | 46.5 | -3.7162 | 334 |
| L aHPC | Parietal_Sup_L | -25.5 | | -61.5 | 57 | -3.529 | 144 |
| L aHPC | Precentral_R | 43.5 | | -12 | 61.5 | -4.0192 | 870 |
| L pHPC | Temporal_Pole_Sup_L | -37.5 | | 12 | -21 | 3.4499 | 445 |
| L pHPC | Hippocampus_L | -28.5 | | -13.5 | -13.5 | 4.1336 | 398 |
| L pHPC | Lingual_L | -24 | | -54 | -3 | 4.2308 | 109 |
| L pHPC | Temporal_Mid_R | 48 | | -58.5 | 13.5 | 4.5496 | 412 |
| L pHPC | Temporal_Sup_R | 57 | | -33 | 22.5 | -3.4199 | 313 |
| L pHPC | Precuneus_R | 7.5 | | -57 | 34.5 | 3.805 | 292 |
| L pHPC | Precentral_R | 16.5 | | -27 | 73.5 | -4.3176 | 565 |
| R aHPC | Temporal_Sup_R | 57 | | -31.5 | 15 | -4.1619 | 206 |
| R pHPC | Hippocampus_L | -30 | | -12 | -13.5 | 3.8223 | 161 |
| R pHPC | Temporal_Pole_Sup_R | 57 | | 15 | -12 | 3.3073 | 134 |
| R pHPC | Temporal_Mid_R | 49.5 | | -60 | 13.5 | 3.9456 | 275 |
| R pHPC | Temporal_Sup_R | 58.5 | | -31.5 | 16.5 | -3.4825 | 173 |
| R pHPC | Parietal_Inf_L | -36 | | -48 | 39 | -3.7025 | 133 |
| R pHPC | Precentral_R | 15 | | -27 | 75 | -4.0688 | 265 |

Abbreviations: L, left; R, right; aHPC, anterior hippocampus; pHPC, posterior hippocampus; NC, normal control; aMCI, amnestic mild cognitive impairment.

**Supplementary Table 18.** Significant between-group(NC-AD) differences in structural covariance networks seeding from anterior and posterior hippocampus.

| seed | AAL region | MNI coordinates | | | Peak intensity | Extent |
| --- | --- | --- | --- | --- | --- | --- |
|  |  | X | Y | Z |  |  |
| L aHPC | Temporal_Mid_L | -66 | -12 | -13.5 | -3.7517 | 174 |
| L aHPC | Lingual_R | 9 | -91.5 | -7.5 | 3.3008 | 152 |
| L aHPC | Frontal_Inf_Tri_L | -49.5 | 28.5 | -1.5 | -3.7935 | 253 |
| L aHPC | Frontal_Inf_Orb_R | 54 | 33 | -4.5 | -3.2883 | 107 |
| L aHPC | Temporal_Mid_L | -54 | -48 | 1.5 | -3.7051 | 389 |
| L aHPC | Thalamus_L | -9 | -7.5 | 4.5 | -3.7979 | 289 |
| L aHPC | Temporal_Sup_R | 40.5 | -27 | 10.5 | -4.0872 | 710 |
| L aHPC | Frontal_Sup_Medial_L | -9 | 55.5 | 13.5 | -3.8894 | 167 |
| L aHPC | Precentral_R | 55.5 | 4.5 | 28.5 | 3.4405 | 105 |
| L aHPC | Frontal_Sup_R | 16.5 | 46.5 | 45 | -3.7327 | 121 |
| L aHPC | Parietal_Inf_L | -31.5 | -42 | 49.5 | -3.9214 | 159 |
| L aHPC | Frontal_Sup_L | -18 | 15 | 49.5 | -3.8057 | 140 |
| L aHPC | Precuneus_R | 3 | -48 | 60 | 3.6251 | 154 |
| L aHPC | Frontal_Sup_R | 15 | -3 | 73.5 | -4.0043 | 156 |
| L pHPC | Frontal_Inf_Orb_R | 46.5 | 36 | -4.5 | -3.7896 | 312 |
| L pHPC | Temporal_Mid_L | -48 | -64.5 | 1.5 | 3.4682 | 172 |
| L pHPC | Heschl_L | -36 | -25.5 | 12 | -3.2515 | 210 |
| L pHPC | Temporal_Sup_R | 60 | -31.5 | 10.5 | -3.337 | 113 |
| L pHPC | Temporal_Sup_R | 60 | -18 | 12 | -3.5531 | 156 |
| L pHPC | Angular_L | -52.5 | -69 | 30 | 4.1876 | 150 |
| L pHPC | Occipital_Sup_R | 33 | -76.5 | 43.5 | 3.689 | 165 |
| L pHPC | Frontal_Sup_L | -18 | 16.5 | 49.5 | -3.9886 | 136 |
| L pHPC | Precuneus_R | 4.5 | -49.5 | 67.5 | 3.5404 | 116 |
| L pHPC | Precentral_R | 30 | -22.5 | 70.5 | -3.1194 | 142 |
| L pHPC | Postcentral_R | 15 | -34.5 | 64.5 | -3.4596 | 124 |
| R aHPC | Insula_L | -40.5 | 12 | -12 | 3.7629 | 131 |
| R aHPC | Insula_R | 43.5 | 13.5 | -7.5 | 4.2362 | 147 |
| R aHPC | Temporal_Pole_Sup_R | 57 | 16.5 | -15 | 3.3875 | 104 |
| R aHPC | Thalamus_L | -9 | -7.5 | 6 | -3.7271 | 231 |
| R aHPC | Heschl_L | -36 | -22.5 | 10.5 | -3.4777 | 129 |
| R aHPC | Temporal_Sup_R | 58.5 | -31.5 | 10.5 | -3.9969 | 416 |
| R aHPC | Frontal_Sup_R | 21 | 58.5 | 10.5 | -3.1659 | 188 |
| R aHPC | Frontal_Mid_L | -28.5 | 28.5 | 36 | -4.4319 | 109 |
| R aHPC | Frontal_Sup_R | 21 | 40.5 | 43.5 | -4.0255 | 243 |
| R aHPC | Parietal_Inf_L | -31.5 | -42 | 48 | -3.2822 | 152 |
| R aHPC | Precentral_R | 48 | -3 | 45 | -3.3035 | 112 |
| R aHPC | Frontal_Mid_L | -25.5 | -6 | 49.5 | -3.6861 | 175 |
| R aHPC | Precentral_L | -16.5 | -16.5 | 73.5 | -3.7381 | 145 |
| R pHPC | Frontal_Sup_Orb_L | -13.5 | 57 | -10.5 | -3.5321 | 116 |
| R pHPC | Frontal_Inf_Orb_R | 46.5 | 36 | -4.5 | -3.9972 | 417 |
| R pHPC | ParaHippocampal_R | 33 | -36 | -4.5 | -4.4366 | 110 |
| R pHPC | Heschl_L | -36 | -25.5 | 12 | -3.898 | 885 |
| R pHPC | Temporal_Sup_R | 67.5 | -42 | 13.5 | -3.374 | 192 |
| R pHPC | Temporal_Sup_R | 60 | -18 | 12 | -4.0471 | 212 |
| R pHPC | SupraMarginal_L | -49.5 | -42 | 27 | -3.5325 | 167 |
| R pHPC | Frontal_Mid_L | -28.5 | 25.5 | 39 | -4.1918 | 270 |
| R pHPC | Frontal_Sup_R | 22.5 | 39 | 43.5 | -3.5624 | 126 |
| R pHPC | Frontal_Sup_L | -18 | 16.5 | 49.5 | -4.3995 | 359 |
| R pHPC | Frontal_Mid_R | 45 | -3 | 58.5 | -3.3997 | 301 |
| R pHPC | Precentral_L | -16.5 | -16.5 | 73.5 | -3.765 | 356 |

Abbreviations: L, left; R, right; aHPC, anterior hippocampus; pHPC, posterior hippocampus; NC, normal control; AD, Alzheimer’s disease.

**Supplementary Table 19.** Significant between-group(NC-SCD) differences in structural covariance networks seeding from subregions of the cholinergic basal forebrain.

| seed | AAL region | MNI coordinates | | | | Peak intensity | Extent |
| --- | --- | --- | --- | --- | --- | --- | --- |
|  |  | X | Y | | Z |  |  |
| Ch4p | Occipital_Mid_L | -40.5 | | -72 | 3 | 3.5079 | 126 |
| Ch4p | Temporal_Mid_L | -52.5 | | -51 | 1.5 | -3.5685 | 133 |
| Ch4a-i | Temporal_Mid_R | 58.5 | | -33 | -9 | -3.6114 | 202 |
| Ch4a-i | Temporal_Mid_L | -46.5 | | -58.5 | 4.5 | -3.4246 | 171 |
| Ch4a-i | Cuneus_R | 16.5 | | -79.5 | 37.5 | -3.8998 | 159 |
| Ch3 | ParaHippocampal_L | -30 | | -18 | -24 | -3.763 | 101 |
| Ch3 | Temporal_Mid_R | 60 | | -33 | -7.5 | -4.1422 | 304 |
| Ch3 | Occipital_Inf_R | 39 | | -67.5 | -4.5 | -3.7946 | 155 |
| Ch3 | Temporal_Mid_L | -49.5 | | -54 | 3 | -3.4821 | 233 |
| Ch3 | Frontal_Mid_R | 36 | | 15 | 58.5 | -3.0011 | 114 |
| Ch4al/NSP | Temporal_Inf_L | -52.5 | | -46.5 | -24 | -3.707 | 134 |
| Ch4al/NSP | Temporal_Inf_R | 55.5 | | -52.5 | -25.5 | -3.8688 | 105 |
| Ch4al/NSP | Temporal_Inf_L | -54 | | -58.5 | -21 | -3.6821 | 161 |
| Ch4al/NSP | Temporal_Inf_R | 57 | | -45 | -10.5 | -3.4219 | 114 |
| Ch4al/NSP | Temporal_Mid_L | -52.5 | | -63 | 6 | -4.0957 | 464 |
| Ch4al/NSP | Paracentral_Lobule_R | 10.5 | | -40.5 | 61.5 | -3.4564 | 123 |
| interstitial nuclei | Temporal_Inf_L | -54 | | -58.5 | -21 | -4.1915 | 160 |
| interstitial nuclei | Temporal_Mid_L | -45 | | -60 | 4.5 | -3.3563 | 141 |
| Ch1/2 | ParaHippocampal_L | -30 | | -18 | -22.5 | -4.1796 | 153 |
| Ch1/2 | Temporal_Mid_R | 60 | | -15 | -19.5 | -3.49 | 124 |
| Ch1/2 | Temporal_Mid_R | 58.5 | | -34.5 | -9 | -3.8966 | 322 |
| Ch1/2 | Parietal_Inf_L | -28.5 | | -43.5 | 49.5 | -3.4975 | 106 |
| Ch1/2 | Frontal_Mid_R | 31.5 | | 16.5 | 57 | -3.6872 | 234 |
| Ch1/2 | Frontal_Sup_L | -22.5 | | 19.5 | 63 | -3.6447 | 105 |

Abbreviations: NSP, Nucleus subputaminalis; NC, normal control; SCD, subjective cognitive decline.

**Supplementary Table 20.** Significant between-group(NC-aMCI) differences in structural covariance networks seeding from subregions of the cholinergic basal forebrain.

| seed | AAL region | MNI coordinates | | | | Peak intensity | Extent |
| --- | --- | --- | --- | --- | --- | --- | --- |
|  |  | X | Y | | Z |  |  |
| Ch4p | Temporal_Inf_L | -54 | | -60 | -15 | -3.6369 | 144 |
| Ch4p | Frontal_Inf_Orb_L | -37.5 | | 24 | -16.5 | -3.8226 | 218 |
| Ch4p | Olfactory_R | 4.5 | | 19.5 | -15 | -3.4756 | 435 |
| Ch4p | Frontal_Sup_Orb_L | -31.5 | | 63 | -4.5 | -4.97 | 1084 |
| Ch4p | Frontal_Inf_Orb_R | 33 | | 22.5 | -6 | -3.3572 | 122 |
| Ch4p | Temporal_Sup_R | 55.5 | | -30 | 16.5 | -5.6571 | 1204 |
| Ch4p | Temporal_Mid_L | -52.5 | | -49.5 | 1.5 | -3.7064 | 192 |
| Ch4p | Frontal_Sup_Medial_L | -9 | | 57 | 15 | -3.5114 | 453 |
| Ch4p | Cuneus_L | -4.5 | | -79.5 | 22.5 | -3.5943 | 208 |
| Ch4p | Frontal_Mid_L | -46.5 | | 45 | 18 | -3.776 | 167 |
| Ch4p | SupraMarginal_L | -49.5 | | -40.5 | 33 | -3.6421 | 107 |
| Ch4p | Cingulum_Mid_L | -3 | | 0 | 36 | -3.8664 | 313 |
| Ch4p | Parietal_Inf_L | -28.5 | | -42 | 48 | -4.3045 | 205 |
| Ch4p | Parietal_Inf_R | 40.5 | | -39 | 40.5 | -3.3796 | 116 |
| Ch4p | Precentral_L | -45 | | 1.5 | 45 | -3.2754 | 106 |
| Ch4p | Angular_R | 27 | | -61.5 | 49.5 | -3.484 | 135 |
| Ch4p | Parietal_Sup_L | -19.5 | | -60 | 54 | -3.3244 | 176 |
| Ch4p | Frontal_Sup_R | 13.5 | | -3 | 73.5 | -4.0212 | 148 |
| Ch4a-i | Temporal_Mid_L | -43.5 | | 7.5 | -31.5 | -4.051 | 106 |
| Ch4a-i | Temporal_Sup_R | 55.5 | | -30 | 15 | -5.002 | 276 |
| Ch3 | Temporal_Sup_R | 55.5 | | -30 | 15 | -4.7111 | 233 |
| Ch3 | Frontal_Mid_L | -46.5 | | 45 | 18 | -3.5564 | 100 |
| Ch4al/NSP | Temporal_Sup_R | 57 | | -31.5 | 15 | -4.5368 | 189 |
| Ch4al/NSP | Frontal_Mid_L | -46.5 | | 45 | 18 | -4.033 | 182 |
| Ch4al/NSP | Parietal_Inf_L | -28.5 | | -42 | 48 | -3.3702 | 117 |
| Ch4al/NSP | Parietal_Sup_L | -16.5 | | -54 | 54 | -3.3736 | 136 |
| Ch4al/NSP | Postcentral_R | 27 | | -39 | 64.5 | 3.5874 | 189 |
| interstitial nuclei | Occipital_Inf_L | -52.5 | | -63 | -16.5 | -3.4041 | 115 |
| interstitial nuclei | Thalamus_R | 18 | | -22.5 | 0 | 3.3644 | 103 |
| interstitial nuclei | Pallidum_R | 18 | | 9 | 4.5 | 3.5564 | 161 |
| interstitial nuclei | Temporal_Sup_R | 57 | | -31.5 | 15 | -4.9532 | 275 |
| interstitial nuclei | Frontal_Inf_Oper_L | -48 | | 12 | 27 | -3.5864 | 133 |
| interstitial nuclei | Cingulum_Mid_L | -3 | | -6 | 36 | -3.4651 | 172 |
| interstitial nuclei | Frontal_Mid_R | 43.5 | | 21 | 43.5 | -3.7808 | 240 |
| Ch1/2 | Temporal_Mid_L | -43.5 | | 7.5 | -31.5 | -3.6664 | 115 |
| Ch1/2 | Temporal_Sup_R | 57 | | -30 | 15 | -4.4193 | 164 |

Abbreviations: NSP, Nucleus subputaminalis; NC, normal control; aMCI, amnestic mild cognitive impairment.

**Supplementary Table 21.** Significant between-group(NC-AD) differences in structural covariance networks seeding from subregions of the cholinergic basal forebrain.

| seed | AAL region | MNI coordinates | | | | Peak intensity | Extent |
| --- | --- | --- | --- | --- | --- | --- | --- |
|  |  | X | Y | | Z |  |  |
| Ch4p | Temporal_Inf_L | -55.5 | -58.5 | -21 | | -4.2971 | 180 |
| Ch4p | Frontal_Inf_Orb_L | -52.5 | 28.5 | -3 | | -4.2368 | 661 |
| Ch4p | Temporal_Mid_L | -64.5 | -13.5 | -12 | | -3.5477 | 126 |
| Ch4p | Frontal_Sup_Orb_L | -15 | 63 | -15 | | -4.052 | 255 |
| Ch4p | Frontal_Inf_Orb_R | 54 | 33 | -4.5 | | -4.2437 | 463 |
| Ch4p | Temporal_Mid_L | -57 | -49.5 | 3 | | -4.3808 | 1021 |
| Ch4p | Frontal_Mid_Orb_L | -36 | 51 | -3 | | -3.8035 | 346 |
| Ch4p | Lingual_L | -16.5 | -55.5 | 3 | | -3.0366 | 129 |
| Ch4p | Thalamus_L | -13.5 | -15 | 3 | | -3.5803 | 208 |
| Ch4p | Temporal_Sup_L | -54 | -6 | 6 | | -3.7213 | 281 |
| Ch4p | Frontal_Sup_Medial_L | -9 | 55.5 | 13.5 | | -4.0643 | 633 |
| Ch4p | Temporal_Sup_R | 46.5 | -30 | 9 | | -4.685 | 1488 |
| Ch4p | Cuneus_L | -4.5 | -81 | 22.5 | | -4.0091 | 155 |
| Ch4p | Frontal_Mid_R | 34.5 | 42 | 25.5 | | -3.8156 | 375 |
| Ch4p | Frontal_Mid_L | -28.5 | 30 | 34.5 | | -4.4557 | 581 |
| Ch4p | Occipital_Mid_L | -48 | -73.5 | 24 | | -3.8361 | 102 |
| Ch4p | Occipital_Mid_R | 43.5 | -73.5 | 30 | | -3.9981 | 157 |
| Ch4p | Frontal_Sup_Medial_L | -1.5 | 58.5 | 33 | | -4.0691 | 281 |
| Ch4p | SupraMarginal_L | -52.5 | -45 | 31.5 | | -4.108 | 178 |
| Ch4p | Cingulum_Mid_R | 1.5 | 36 | 31.5 | | -5.0984 | 240 |
| Ch4p | Cingulum_Mid_L | -6 | 1.5 | 37.5 | | -4.0552 | 384 |
| Ch4p | Parietal_Inf_L | -36 | -43.5 | 39 | | -4.3655 | 416 |
| Ch4p | Frontal_Sup_Medial_L | 0 | 45 | 42 | | -3.6915 | 117 |
| Ch4p | Precentral_R | 55.5 | 3 | 46.5 | | -3.3552 | 174 |
| Ch4p | Precentral_L | -36 | -9 | 51 | | -4.3516 | 781 |
| Ch4p | Frontal_Sup_R | 24 | 37.5 | 43.5 | | -3.5659 | 133 |
| Ch4p | Parietal_Sup_R | 24 | -66 | 52.5 | | -3.6758 | 115 |
| Ch4p | Frontal_Sup_L | -18 | 16.5 | 49.5 | | -3.4434 | 202 |
| Ch4p | Parietal_Sup_L | -24 | -61.5 | 58.5 | | -3.802 | 382 |
| Ch4p | Precentral_R | 42 | -9 | 63 | | -3.5734 | 226 |
| Ch4a-i | Frontal_Sup_Orb_L | -15 | 55.5 | -12 | | -3.218 | 100 |
| Ch4a-i | Heschl_R | 37.5 | -22.5 | 13.5 | | -3.3813 | 276 |
| Ch4a-i | Cingulum_Ant_L | -1.5 | 39 | -6 | | -3.6723 | 484 |
| Ch4a-i | Thalamus_L | -1.5 | -18 | 1.5 | | -3.3729 | 289 |
| Ch4a-i | Temporal_Sup_R | 57 | -30 | 10.5 | | -4.3108 | 397 |
| Ch4a-i | Frontal_Mid_L | -28.5 | 30 | 34.5 | | -3.7062 | 162 |
| Ch4a-i | Parietal_Inf_L | -36 | -45 | 39 | | -3.9476 | 224 |
| Ch4a-i | Precentral_L | -39 | 0 | 45 | | -4.201 | 873 |
| Ch4a-i | Parietal_Sup_L | -16.5 | -63 | 58.5 | | -3.1312 | 162 |
| Ch4a-i | Precuneus_R | 3 | -48 | 58.5 | | 3.1733 | 120 |
| Ch4a-i | Frontal_Sup_R | 13.5 | -4.5 | 73.5 | | -3.9269 | 130 |
| Ch3 | Temporal_Mid_L | -64.5 | -15 | -13.5 | | -3.2165 | 161 |
| Ch3 | Cingulum_Ant_L | -1.5 | 39 | -6 | | -3.8335 | 560 |
| Ch3 | Heschl_R | 37.5 | -21 | 12 | | -3.7829 | 570 |
| Ch3 | Thalamus_L | -13.5 | -16.5 | 1.5 | | -3.2956 | 160 |
| Ch3 | Temporal_Sup_R | 58.5 | -31.5 | 10.5 | | -4.3081 | 324 |
| Ch3 | Temporal_Sup_R | 63 | -15 | 12 | | -3.8372 | 142 |
| Ch3 | Frontal_Mid_L | -30 | 30 | 36 | | -3.8769 | 223 |
| Ch3 | Parietal_Inf_L | -36 | -45 | 39 | | -3.8454 | 211 |
| Ch3 | Precentral_L | -37.5 | 0 | 45 | | -3.8876 | 381 |
| Ch3 | Frontal_Mid_L | -24 | 4.5 | 63 | | -3.7238 | 249 |
| Ch3 | Precuneus_R | 1.5 | -49.5 | 57 | | 3.2869 | 134 |
| Ch3 | Parietal_Sup_L | -16.5 | -57 | 60 | | -3.2697 | 153 |
| Ch4al/NSP | Temporal_Sup_R | 45 | -19.5 | 1.5 | | -3.3132 | 237 |
| Ch4al/NSP | Precentral_L | -45 | -7.5 | 51 | | -3.4773 | 166 |
| Ch4al/NSP | Parietal_Sup_L | -18 | -57 | 60 | | -3.6489 | 175 |
| Ch4al/NSP | Frontal_Sup_L | -24 | 4.5 | 64.5 | | -3.2104 | 102 |
| interstitial nuclei | Caudate_R | 15 | 10.5 | 10.5 | | 3.4308 | 347 |
| interstitial nuclei | Temporal_Sup_R | 66 | -16.5 | 12 | | -3.262 | 118 |
| interstitial nuclei | Temporal_Sup_R | 43.5 | -21 | 1.5 | | -3.934 | 427 |
| interstitial nuclei | Temporal_Mid_R | 48 | -64.5 | 19.5 | | 3.5295 | 118 |
| interstitial nuclei | Parietal_Inf_L | -36 | -45 | 40.5 | | -3.5868 | 172 |
| interstitial nuclei | Precentral_L | -39 | 0 | 45 | | -3.3468 | 239 |
| interstitial nuclei | Precuneus_R | 4.5 | -48 | 57 | | 4.1277 | 324 |
| interstitial nuclei | Parietal_Sup_L | -24 | -63 | 58.5 | | -3.8021 | 175 |
| interstitial nuclei | Precentral_L | -18 | -16.5 | 73.5 | | -3.7238 | 100 |
| interstitial nuclei | Supp_Motor_Area_R | 13.5 | -4.5 | 72 | | -3.8631 | 196 |
| Ch1/2 | Temporal_Pole_Sup_R | 55.5 | 16.5 | -13.5 | | 3.8021 | 171 |
| Ch1/2 | Calcarine_R | 7.5 | -91.5 | 0 | | 3.3761 | 116 |
| Ch1/2 | Precentral_L | -39 | -1.5 | 43.5 | | -4.1167 | 416 |
| Ch1/2 | Parietal_Sup_L | -16.5 | -63 | 61.5 | | -3.8936 | 134 |
| Ch1/2 | Precentral_R | 42 | -9 | 61.5 | | -3.1172 | 143 |
| Ch1/2 | Frontal_Sup_L | -24 | 6 | 64.5 | | -4.2794 | 205 |
| Ch1/2 | Precentral_L | -16.5 | -16.5 | 73.5 | | -4.2474 | 263 |

Abbreviations: NSP, Nucleus subputaminalis; NC, normal control; AD, Alzheimer’s disease.

**Supplementary Table 22.** Partial correlation analyses between peak cluster volumes and the scores of neuropsychological tests in individuals with SCD.

| Seed | Region | contrast | MoCA | AVLT_I | AVLT_D | AVLT_R |
| --- | --- | --- | --- | --- | --- | --- |
| L aHPC | Temporal_Mid_L | NC>SCD | 0.016(0.931) | -0.245(0.176) | -0.193(0.290) | **-0.351(0.049)** |
| L pHPC | Temporal_Mid_R | NC>SCD | 0.119(0.517) | **0.380(0.032)** | 0.310(0.084) | **0.505(0.003)** |
| R aHPC | SupraMarginal_R | NC>SCD | -0.001(0.996) | -0.106(0.563) | -0.092(0.615) | 0.289(0.109) |
|  | Occipital_Mid_L | NC<SCD | 0.254(0.160) | 0.172(0.345) | 0.025(0.890) | -0.029(0.208) |
| R pHPC | Frontal_Sup_L | NC>SCD | -0.279(0.123) | -0.121(0.511) | -0.206(0.259) | 0.117(0.523) |
| Ch4p | Temporal_Mid_L | NC>SCD | 0.045(0.86) | -0.204(0.262) | -0.156(0.395) | -0.261(0.150) |
|  | Occipital_Mid_L | NC<SCD | 0.230(0.205) | 0.151(0.409) | -0.070(0.702) | -0.248(0.172) |
| Ch1/2 | ParaHippocampal_L | NC>SCD | 0.159(0.385) | 0.197(0.281) | 0.158(0.387) | 0.079(0.666) |
| R EC | SupraMarginal_R | NC>SCD | -0.119(0.518) | -0.228(0.209) | -0.164(0.369) | -0.282(0.118) |
| L PCC | Temporal_Inf_R | NC>SCD | -0.137(0.445) | 0.075(0.685) | -0.266(0.141) | 0.028(0.878) |
|  | Precuneus_R | NC<SCD | 0.155(0.397) | 0.003(0.986) | 0.140(0.444) | -0.147(0.421) |
| R FIC | Parietal_Inf_L | NC>SCD | -0.120(0.512) | 0.161(0.380) | 0.011(0.952) | -0.050(0.786) |
| R DLPFC | Temporal_Inf_R | NC>SCD | .101(0.582) | -0.049(0.789) | -0.158(0.388) | -0.068(0.712) |
|  | Occipital_Mid_L | NC<SCD | 0.154(0.399) | 0.005(0.978) | -0.195(0.284) | -0.294(0.102) |

The values are the correlation coefficients and p values in parentheses.

Abbreviations: L, left; R, right; aHPC, anterior hippocampus; pHPC, posterior hippocampus; EC, entorhinal cortex; PCC, posterior cingulate cortex DLPFC, dorsolateral prefrontal cortex; FIC, frontoinsular cortex; AVLT, auditory verbal learning test; AVLT_I, AVLT-immediate recall; AVLT_D, AVLT-delayed recall; AVLT_R, AVLT-recognition MoCA, Montreal Cognitive Assessment; NC, normal controls; SCD, subjective cognitive decline

**Supplementary Table 23.** Partial correlation analyses between peak cluster volumes and the scores of neuropsychological tests in aMCI patients.

| Seed | Region | contrast | MoCA | AVLT_I | AVLT_D | AVLT_R |
| --- | --- | --- | --- | --- | --- | --- |
| L aHPC | Precentral_R | NC>aMCI | -0.170(0.295) | 0.141(0.385) | 0.219(0.175) | 0.154(0.343) |
|  | Hippocampus_L | NC<aMCI | 0.239(0.138) | 0.219(0.174) | **0.351(0.027)** | **0.456(0.003)** |
| L pHPC | Precentral_R | NC>aMCI | -0.265(0.099) | 0.088(0.590) | 0.002(0.990) | -0.079(0.626) |
|  | Temporal_Mid_R | NC<aMCI | 0.030(0.856) | -0.037(0.819) | 0.159(0.326) | **0.420(0.007)** |
| R aHPC | Temporal_Sup_R | NC>aMCI | -0.076(0.640) | -0.161(0.320) | -0.152(0.350) | -0.202(0.211) |
| R pHPC | Precentral_R | NC>aMCI | -0.286(0.073) | 0.066(0.686) | -0.034(0.834) | -0.110(0.501) |
|  | Temporal_Mid_R | NC<aMCI | 0.09(0.580) | -0.007(0.967) | 0.206(0.202) | **0.463(0.003)** |
| Ch4p | Temporal_Sup_R | NC>aMCI | -0.219(0.175) | **-0.345(0.029)** | -0.145(0.374) | -0.216(0.181) |
| Ch1/2 | Temporal_Sup_R | NC>aMCI | -0.133(0.412) | -0.164(0.311) | -0.113(0.487) | -0.209(0.195) |
| R EC | Precentral_L | NC>aMCI | 0.149(0.358) | 0.037(0.823) | 0.058(0.722) | 0.040(0.805) |
| L PCC | Frontal_Mid_L | NC>aMCI | 0.083(0.612) | 0.203(0.210) | 0.137(0.399) | 0.159(0.328) |
|  | Temporal_Mid_R | NC<aMCI | -0.011(0.946) | 0.039(0.811) | 0.157(0.332) | **0.401(0.010)** |
| R FIC | Precentral_R | NC>aMCI | **-0.318(0.045)** | -0.018(0.910) | -0.032(0.847) | -0.119(0.463) |
|  | Temporal_Mid_R | NC<aMCI | 0.152(0.350) | 0.200(0.217) | 0.207(0.199) | **0.376(0.017)** |
| R DLPFC | Cingulum_Mid_R | NC<aMCI | -0.007(0.964) | 0.073(0.654) | 0.055(0.737) | 0.169(0.296) |

The values are the correlation coefficients and p values in parentheses.

Abbreviations: L, left; R, right; aHPC, anterior hippocampus; pHPC, posterior hippocampus; EC, entorhinal cortex; PCC, posterior cingulate cortex; DLPFC, dorsolateral prefrontal cortex; FIC, frontoinsular cortex; AVLT, auditory verbal learning test; AVLT_I, AVLT-immediate recall; AVLT_D, AVLT-delayed recall; AVLT_R, AVLT-recognition MoCA, Montreal Cognitive Assessment; NC, normal controls; aMCI, amnestic mild cognitive impairment

**Supplementary Table 24.** Partial correlation analyses between peak cluster volumes and the scores of neuropsychological tests in AD patients.

| Seed | Region | Contrast | MoCA | AVLT_I | AVLT_D | AVLT_R |
| --- | --- | --- | --- | --- | --- | --- |
| L aHPC | Temporal_Sup_R | NC>AD | 0.111(0.509) | 0.163(0.329) | 0.014(0.932) | -0.097(0.562) |
|  | Precuneus_R | NC<AD | 0.194(0.242) | 0.288(0.080) | **0.450(0.005)** | 0.151(0.364) |
| L pHPC | Frontal_Sup_L | NC>AD | 0.247(0.135) | 0.247(0.135) | 0.066(0.695) | -0.074(0.661) |
|  | Angular_L | NC<AD | 0.275(0.095) | 0.306(0.062) | 0.248(0.133) | 0.043(0.798) |
| R aHPC | Frontal_Mid_L | NC>AD | 0.314(0.055) | **0.405(0.012)** | 0.264(0.109) | -0.008(0.962) |
|  | Insula_R | NC<AD | 0.029(0.864) | 0.099(0.055) | 0.239(0.149) | 0.079(0.638) |
| R pHPC | ParaHippocampal_R | NC>AD | 0.293(0.074) | 0.197(0.235) | 0.207(0.213) | 0.153(0.358) |
| Ch4p | Cingulum_Mid_R | NC>AD | 0.239(0.148) | 0.149(0.373) | 0.099(0.554) | 0.123(0.462) |
| Ch1/2 | Frontal_Sup_L | NC>AD | 0.279(0.090) | 0.216(0.193) | 0.198(0.285) | 0.104(0.534) |
|  | Temporal_Pole_Sup_R | NC<AD | 0.206(0.214) | -0.002(0.992) | 0.257(0.119) | 0.199(0.232) |
| R EC | Precentral_L | NC>AD | 0.150(0.369) | 0.264(0.110) | 0.307(0.060) | 0.049(0.796) |
|  | ParaHippocampal_R | NC<AD | 0.048(0.775) | -0.046(0.786) | 0.284(0.084) | 0.080(0.631) |
| L PCC | Precentral_L | NC>AD | 0.148(0.375) | 0.147(0.379) | 0.213(0.198) | 0.135(0.419) |
|  | Temporal_Mid_R | NC<AD | 0.253(0.125) | 0.212(0.201) | 0.219(0.186) | 0.060(0.722) |
| R FIC | Precentral_R | NC>AD | -0.289(0.078) | -0.162(0.332) | 0.159(0.342) | -0.198(0.232) |
|  | Supp_Motor_Area_R | NC<AD | 0.259(0.117) | **0.397(0.014)** | 0.286(0.081) | 0.257(0.120) |
| R DLPFC | Calcarine_L | NC>AD | 0.127(0.448) | 0.035(0.833) | -0.123(0.463) | 0.118(0.481) |
|  | Precuneus_R | NC<AD | **0.321(0.049)** | **0.319(0.051)** | 0.202(0.224) | 0.068(0.683) |

The values are the correlation coefficients and p values in parentheses.

Abbreviations: L, left; R, right; aHPC, anterior hippocampus; pHPC, posterior hippocampus; EC, entorhinal cortex; PCC, posterior cingulate cortex DLPFC, dorsolateral prefrontal cortex; FIC, frontoinsular cortex; AVLT, auditory verbal learning test; AVLT_I, AVLT-immediate recall; AVLT_D, AVLT-delayed recall; AVLT_R, AVLT-recognition MoCA, Montreal Cognitive Assessment; NC, normal controls; AD, Alzheimer’s disease
